# Reducing COVID-19 risk in schools: a qualitative examination of secondary school staff and family views and concerns in the South West of England

**DOI:** 10.1136/bmjpo-2020-000987

**Published:** 2021-03-10

**Authors:** Ava Lorenc, Joanna May Kesten, Judi Kidger, Rebecca Langford, Jeremy Horwood

**Affiliations:** 1 Population Health Sciences, University of Bristol, Bristol, Bristol, UK; 2 NIHR Applied Research Collaboration West, University Hospitals Bristol and Weston NHS Foundation Trust, Bristol, Bristol, UK; 3 NIHR Health Protection Research Unit (HPRU) in Behavioural Science and Evaluation, University of Bristol, Bristol, Bristol, UK

**Keywords:** COVID-19

## Abstract

**Objective:**

To investigate student, parent/carer and secondary school staff attitudes towards school COVID-19 mitigation measures.

**Methods:**

Recruitment used school communication, community organisations and snowball sampling in South West England. Audio recorded online or phone individual/group semi-structured interviews were conducted in July–Septtember 2020 and lasted 30–60 min. Interviews focused on views towards social distancing, hand hygiene and testing. Framework analysis was performed on interview notes/transcripts.

**Results:**

Participants were 15 staff, 20 parents and 17 students (11–16 years) from 14 diverse schools. Concerns about COVID-19 risk at school, especially to vulnerable individuals, were outweighed by perceived risks of missed learning. Some staff felt guilt around being a potential ‘spreader’ by teaching multiple classes. Findings highlighted a wide variety of school COVID-19 mitigation measures being deployed due to ambiguous government guidance. Participants generally saw mitigation measures as an acceptable and pragmatic solution to the perceived impossibility of social distancing in crowded schools, although anticipated challenges changing habitual behaviour. Participants supported school COVID-19 testing but identified the need to consider data security and stigma around COVID-19 diagnosis. Staff were concerned about unintended consequences of risk-reduction strategies on student behaviour, learning and pastoral care, particularly for those with Special Educational Needs or mental health issues who may find the measures especially challenging, and resultant widening inequalities.

**Conclusion:**

Families and staff supported COVID-19 mitigation measures in schools and would welcome the roll out school COVID-19 testing. Clear messaging and engendering collective responsibility are important for compliance and success of COVID-19 mitigation measures. However, schools and policy-makers should consider unintended consequences of measures, providing extra support for vulnerable students and those with additional needs, and consider ways to avoid widening educational and health inequalities. Findings demonstrate the acceptability of school COVID-19 infection control measures is likely to be influenced by the balance of risks and benefits to students.

**What is known about the subject**

- Schools reopened in September 2020 with COVID-19 risk reduction measures in place. Compliance with and impact of these measures—especially on vulnerable groups—is unclear.
- Current UK government COVID-19 policy includes punitive measures to ensure behavioural compliance.
- Scientists have advised scaling-up testing and tracing within schools.

**What this study adds**

- Given the challenges of social distancing under current government school guidelines, families and staff view effective testing and innovative risk reduction measures as imperative.
- Schools should adopt an encouraging and compassionate approach to implementing risk reduction measures, with clear messaging emphasising collective responsibility.
- Families and staff support schools remaining open but concerns over unintended consequences of risk reduction measures must be addressed by schools and policy-makers.

## Introduction

To reduce the spread of COVID-19, a UK ‘lockdown’ was announced on 23 March 2020 and school campuses, as in most countries worldwide,^1^ were closed to all but vulnerable or priority students. Prior to the summer holidays some year groups, including year 10, returned to school for face-to-face teaching. In July, the government announced all school campuses would fully reopen in September.

Evidence for school closures reducing infection spread is equivocal.^2^ Those in favour of school campuses reopening to all pointed to the impact on learning,^3^ particularly for lower socioeconomic groups,^4^ widening inequalities and consequences for students’ physical and mental health.^1 5–10^ Remote learning also affected staff well-being and mental health.^11^ However, schools reopening carried risks modelling suggested increased COVID-19 infection would occur if schools reopened full time (combined with easing of other restrictions and without scaled up school testing).^12^

Government guidance set out a range of school-based protective measures to reduce COVID-19 outbreaks, including hand hygiene, cleaning, reporting symptoms and social distancing. Further, a UK modelling study recommended existing testing should be scaled up.^12 13^

Understanding the views and concerns of school staff, parents and students about the implementation of this guidance is key to ensuring compliance, avoiding unintended harms^13^ and supporting schools to reduce risks. This is especially important in secondary schools as the risk of transmission in teenagers is higher than young children, COVID-19 measures particularly impact examination years, and secondary school students are more concerned than primary about COVID-19.^14^ This qualitative study undertaken in secondary schools aimed to rapidly explore student, parent/carer and school staff attitudes towards school COVID-19 mitigation measures, views on managing COVID-19 infections in schools and opinions about student groups who may be particularly affected by these measures.

## Methods

### Study setting

The study was conducted during the school summer holidays immediately following school campus closures and the first week of the new academic year (between July and September 2020), with 11–16 years, parents and school staff recruited via secondary schools and local community organisations in the South West of England.

### Sampling and recruitment

Because lower socioeconomic and Black, Asian and Minority Ethnic (BAME) populations are disproportionately affected by COVID-19, we contacted 21 secondary schools with relatively higher levels of these populations (based on school-level data from local Public Health departments) to ensure we captured their specific concerns. Participating schools sent study information to potential participants (eg, by email/newsletter). We included staff (teachers, senior leaders/headteachers, Special Educational Needs Coordinators (SENCOs), or those with a role in infection control), students and their parents/carers, at Bristol secondary state schools. We focused student/parent recruitment on year 8 (age 12–13; most had no face-to-face teaching since March) and year 10 (age 14–15; had briefly returned to school campus prior to the summer holidays; faced key exams next year). We also advertised the study via community organisations (eg, BAME support groups, youth groups) in areas of higher BAME groups/deprivation (newsletters, social media and direct contact with members) and used snowball sampling (including families inviting friends to be interviewed with them or separately). Individuals interested in participating contacted the researcher directly to arrange an interview. All eligible volunteers were interviewed (none refused or dropped out).

### Interviews

Participants chose to be interviewed on their own, with a friend and/or parent (students) or with colleagues (staff). AL (an experienced qualitative researcher) led interviews by phone or video, with JMK or JK present for group interviews/student interviews without a parent present. Interviews were 30–60 min and audio recorded. Participants provided audio-recorded verbal informed consent (or assent and parent consent for those under 16 years).

Students and school staff provided input into the study design and topic guides.

Topic guides (see online supplemental materials) were tailored for staff or families, but both covered attitudes towards UK recommendations at the time (social distancing, school hand hygiene and infection control strategies) and acceptability of school COVID-19 testing, and were used flexibly, allowing exploration of issues raised by participants. We offered participants a shopping voucher as thanks for their time (£10 for each family member; £20 for staff).

10.1136/bmjpo-2020-000987.supp1 Supplementary data

Data collection was informed by the concept of information power^15^ and pragmatic considerations of the project timeline.

### Patient and public involvement

The National Institute for Health Research Applied Research Collaboration West Young Peoples Advisory Group provided feedback on study design and interview topic guides.

### Analysis

Producing timely reports for local and national stakeholders necessitated rapid analysis (reports available at https://arc-w.nihr.ac.uk/research/projects/the-back-to-school-study). The framework method^16^ was used to analyse the data. AL used anonymised interview notes to develop an initial coding framework in Microsoft EXCEL. Codes reflecting the topic guide headings and inductive coding were combined to produce overarching themes. Data within each code were summarised. The framework was further developed using verbatim anonymised interview transcripts. Each author independently read through a subset of interviews and added new codes and quotes to the existing framework, discussing these additions as a team. Framework analysis was appropriate for the specific a priori questions and limited time frame.

## Results

### Participants

Three of the 21 contacted schools participated by sending information to potential participants, with participants from an additional 11 schools recruited via other methods. We did not record how participants heard about the study. Participants were 15 school staff (heads/assistant heads, teachers, SENCOs) from 7 schools, and 20 families from 10 schools (12 with a BAME child/parent)—17 students (9 females; 8 males; 6 years 7 s, 4 years 8 s, 2 years 9 s, 5 years 10 s) and 20 parents (19 mothers, 1 father) (table 1). For family interviews, nine were one parent and one child (one where child was interviewed alone so an additional researcher was present), six one parent only, three one parent and two children, and one was two families together (friends and one parent each). All 5 years 10 students had attended school for a few days, and all staff participants had been working during lockdown (some of whom had worked within the school environment).

**Table 1:** Participant characteristics

School	Deprivation quintile (IMD 2019)† (1=least, 5 most)	% of students on free school meals (2020)	% of students with English as an additional language (2018/19)	Families (n)	Staff (n)
S1	2	Under 10	20–30	3	5
S2	5	30–40	40–50	1	3
S3	3	10–20	30–40	8*	2
S4	3	20–30	20–30	3	0
S5	2	10%–20%	Under 10	0	2
S6	1	10–20	10–20	0	1
S7	2	Under 10	20–30	1	0
S8	1	10–20	Under 10	0	1
S9	5	Under 10	Under 10	1	0
S10	3	10–20	Under 10	1	0
S11	1	10–20	Under 10	0	1
S12	4	10–20	10–20	1	0
S13	1	10–20	10–20	1	0
S14	1	Under 10	Under 10	1*	0
Totals				20 (17 students, 20 parents)	13

*One family had children at both these schools.

† Index of Multiple Deprivation (IMD)

### COVID-19 risk concerns

Most staff and around half of students and parents (hereafter referred to as families where there is agreement in views) were not concerned about personal/family COVID-19 risk from returning to school (table 2). However, many staff anticipated increased cases describing schools as ‘petri dishes’. Some staff worried about being a potential ‘spreader’ (table 2). Staff also had concerns about higher risk staff and students, including those from BAME backgrounds, those who were pregnant or who had health conditions.

**Table 2:** Quotes on concern about risk of COVID-19 (using pseudonyms and S: school)

Not concerned about COVID-19/risk outweighed by risk of school campuses not reopening	Concerns about COVID-19
‘I’m not worried about spreading it…I don’t really come in contact with anyone who would be worried’ (Emma, yr 8, S1) ‘I’m not worried about illness(Covid-19), per se, I don’t think…That’s not something that worries me. I’d rather they got… got back to normal, to be honest’ (Jess, Mum, S3) ‘if you want the kids back at school, you, you have to accept that… there are risks associated with that’ (Georgina, Mum, S3) ‘Resigned acceptance [about school opening] that, that the world has changed and, um, that we kind of just have to get on with things’ (Anna, SENCO, S1)	‘I’m worried about um—because Jo’s Dad is in the kind of very, what’s the word I’m looking for? Vulnerable, like medical category ‘cause he’s only got one functioning lung and my Dad is…has dementia so I’m worried about [Jo] bringing coronavirus home’ (Kelly, Mum, S4) ‘I’m a bit concerned. Um, what precaution they’re going to take and as Asian background we come on the high risk’ (Fatima, Mum, S10) ‘I still would feel awful if I got my partner really ill, you know, um, from my work, when he’s allowed to work from home and he’s, you know, following all the guidance’ (Clare, Teacher, S8) ‘teachers that I’ve spoken to that have expressed anxieties are teachers who, you know, with things like low-level asthma and, er, other conditions that put them in a position where they may not be COVID fit’ (John, Teacher, S3)

Many families were more concerned about the negative educational consequences of students not being at school, accepting that home schooling cannot continue indefinitely. However, families did note the risk to vulnerable family members (due to health or age) (table 2). Concern was more common in families with BAME members, although a minority explicitly cited ethnicity-related risk, with some noting the lack of scientific understanding around this risk.

### School risk reduction measures

Outside school, most students had accepted social distancing, wearing masks and handwashing, understanding their necessity, with minor negative comments such as social distancing being ‘not nice’. Most aimed to socially distance and some had almost no social contact outside their household during the first lockdown, some enjoying staying home.

Interviewees highlighted a wide variety of school COVID-19 risk reduction measures (see box 1). Participants agreed such measures were needed, although staff were frustrated with the lack of detail in government guidance, meaning each school had to develop their own plans. Staff were generally happy with schools’ plans but had reservations about feasibility and how they would ‘play out in practice’.

Box 1 **Anticipated risk reduction measures at school**

**The most common measures planned were:**

‘Bubbles’: Groups of students (most commonly year groups) prevented from mixing with other bubbles, and often contained in one part of the school. Separate lunch and break times for bubbles.

Social distancing: 2 m distancing between teachers and students. Distancing not expected between students.

Reduced movement of students around school: students staying in one classroom and teachers moving between classrooms, staggered arrival and leaving, one-way systems, desks facing forward.

Hand sanitiser gel: available throughout school. Cleaning: extra cleaning/facilitating cleaning (eg, removing soft furnishings).

**Less common were:**

Reduced group sessions: no/reduced assemblies or tutor groups sessions (where these were ‘vertical’, ie, included multiple year groups).

Reduced range of subjects: suspending or adapting high COVID-19 risk lessons/activities for example, music, cooking, science experiments, field trips.

Handwashing reminders: handwashing at certain times of day and posters/reminders to wash hands.

Increased ventilation

Handwashing facilities: Outdoor sinks.

Masks: Rules on wearing face coverings varied as the government guidance changed during data collection, but latterly schools required face coverings to be worn outside of classrooms.

### Barriers/concerns regarding risk reduction measures

All participants agreed student social distancing was impossible and ‘pointless’ given the numbers of people and lack of space (table 3). Year ‘bubbles’ (see Box 1) were seen as a ‘pragmatic solution’, although there was concern about crossover via siblings or groups leaving school together, and teachers (though less commonly reported). In terms of compliance with risk reduction measures, of most concern among families was forgetting, and a desire to ‘be normal’. A few staff were concerned about rule enforcement methods.

**Table 3:** Quotes about school COVID-19 risk reduction measures (using pseudonyms and S: school)

Concerns/barriers	Facilitators
Social distancing impossible	Common good
‘I can't see it [social distancing] happening unless there are less children in the school at one time’ (Sarah, Mum, S4) ‘you can’t properly socially distance in a school so it’s a lot of 'that’ll do, that’ll do' but it’s still best it can be’ (Matt, Teacher, S3) ‘maybe they [school] have some magical plan, or maybe if they can extend something or something like that, but I… I just feel like there’s not enough space for what they’re trying to do’ (Amber, yr 7, S3)	‘I think empowering people to be autonomous and realise that their efforts are, are for the collective good. That they’ve all got a part to play in it. I think that’s much better than, you know, er, publicly shaming them, or or, or criticising them…if you want to achieve compliance and cooperation, it’s creating an atmosphere that it’s in everyone’s best interest and you contribute to the common good’ (John, Teacher, S3)
Concerns about year bubbles	Messaging and reminders
‘at the end of the day, we’re all gonna walk out of school at the same time and we’re all just gonna walk together and end up in the same place’ (Amber, yr 7, S3) ‘siblings they are mixing bubbles, the bubbles become 500, 700 [people], um, so it’s a gesture’ (Claire, Teacher, S8)	‘if there’s a consistent message, 'this is just what you do now, if you can', then, um, then they’ll just pick it up really and get on with it’ (Georgina, Mum, S3) ‘I think having more visuals is really quite key so there’s, they are reminded of what’s, what’s going on. Um, I don’t feel like we have enough at the moment, to be honest’ (Julia, Teacher, S11)
Concerns about hand-hygiene	Staff culture shift
‘I suspect we’re going to run out of hand sanitisers in the, um, the ones that are kind of communally put up around the school …I’d say that they, they’re going to run out by break time’ (Julia, Teacher, S11) ‘They [school bathrooms] never have soap or anything, like, loads of the taps don’t work!’ (Daisy, yr 7, S3)	‘our biggest weapon will be ‘don’t be in work if you’re ill’ (Jackie, Head, S1)
Lack of compliance with new rules	
‘[on handwashing] maybe forgetting or teachers not reminding the students enough or them being too lazy or maybe their friends might be going somewhere but their friends don’t wanna wait for them so they just can’t be bothered to wash their hands’ (Ali, yr 10, S13) ‘I feel like, you know, people are just are not gonna do it [social distance]. They’re just gonna do what they want and like run around the whole place’ (Amber, yr 7, S3) ‘a line that’s been put on the floor that shows the two metres to the teacher. Um, what’s interesting there, of course some students are going to see that as a bit of an invite or a challenge to cross that line, literally’ (Tracey, Mum, S3) ‘[my child] might just go to the sink, wash the few fingers at the front and then leave. We don’t actually know if they’re going to be doing the full happy birthday… it baffles me how it’s [enforcing handwashing] actually going to work” (Samira, Mum, S4)	
Specific groups	
‘disadvantaged students, and students that would normally get more support in a lesson - because it’s more like lecture style now, so we’re not really allowed to walk between the desks or anything—those students are going to miss out, the ones who wouldn’t normally put their hand up’ (Finn, Teacher, S5) ‘the young people … with the highest level of needs, who’ve got education health and care plans …might not fully understand the need for personal space, or…we’ve just added this whole extra kind of layer of complexity into their social world that I’m sure is going to be hugely challenging’ (Anna SENCO, S1)	

Staff and family concerns about hand-hygiene/infection control were mostly practical, including: availability of resources (sanitiser/soap, sinks, cleaners)—one school estimated a £40 k cost of hand sanitiser; bathroom cleanliness; time for handwashing and effective use of measures, for example, hand sanitiser versus washing, or proper use of masks. Some were concerned about ventilation for example, windows not opening.

A minority of staff were worried about behavioural issues arising from students having to stay in the same classroom for example, unsupervised lesson changeovers, student boredom and lack of movement. Other concerns included risk of using public transport and reduced range of lessons/activities. An important concern for staff was the impact of these measures on learning and pastoral care, especially social distancing measures, for example, fewer interactive lessons and less opportunity to support individual students.

Staff were concerned social distancing measures would particularly affect students with special educational needs (SEN) or mental health issues. Particular issues for students with SEN included (table 3): struggling to understand and comply with the changes; finding less interactive lessons challenging; SEN staff being unable to work physically closely with students (and lack of Personal Protective Equipment (PPE) for this); physical needs for example, personal care or feeding; and removal of ‘safe spaces’ (due to bubbles and infection risk from soft furnishing).

### Facilitators/suggestions regarding risk reduction measures

The main suggestion from staff for facilitating the new rules was educating students about their importance, and encouraging a ‘we culture’ of collective responsibility through a supportive, considerate approach (table 3), although a minority of families thought handwashing should be compulsory and enforced.

Staff and parents suggested clear consistent messaging and daily reminders, both verbal and visual. Clear and regular communication from schools about the measures would also reassure parents (table 3).

Other staff suggestions to support the mitigation measures included:

- Funding for cleaning products, PPE and hand-sanitiser; additional classroom equipment to ensure no sharing.
- Government guidance on PPE and students with SEN.
- Staff training on how to work within the new measures.
- Risk assessments for vulnerable students/staff.
- Shorter lessons to allow time for handwashing.
- Students bringing their own hand-sanitiser.
- Encouraging staff to stay home if displaying symptoms (a culture shift from presenteeism common in schools) (table 3).

### School management of COVID-19 cases

#### Reaction to COVID-19 cases

Several families, mostly BAME families, anticipated possible stigma around COVID-19 diagnosis. Staff did not generally anticipate stigma, due to increasing understanding of COVID-19 among students, and school communities having a generally tolerant and accepting attitude due to existing school population diversity—although two mentioned possible ‘mass hysteria’ and another that teenagers ‘love to joke and point a finger’ (see box 2).

Box 2 **Quotes about school management of COVID-19 cases**

**Reactions to COVID-19 cases**

‘if someone coughed in my class, I would see one or two people shying away from them a bit and that most of the class is laughing a little bit and saying, ‘oh you have Corona virus’ as a joke’ (David, yr 8, S3)

‘I think everyone would just be very pragmatic about it and I can’t see there being panic stations’ (John, Teacher, S3)

**Reporting symptoms to school**

‘I think it will be very tricky… for parents to differentiate… fever and cough, they are quite common symptoms kids get in the winter season’ (Sangita, Mum, S13)

‘People may also not want to tell them about symptoms due to like embarrassment and stuff so if they—everyone finds out that they have symptoms’ (Jasmine, yr 10, S4)

**School COVID-19 testing**

‘If there was a risk we were going to get the virus and it would make everyone safer then I would do it [(regular testing])’ (Jasmine, yr 10, S4)

‘I think it’s good to see if people might have the coronavirus every month’ (Lily, yr 8, S3)

‘I understand the need [(to collect data]) but on the same token, it’s just that personal data being collected about my child makes me feel very uncomfortable’ (Sarah, mum, S4)

‘market it towards the fact that they’ve missed so much education, this is something that can potentially help keep them in school for as long as possible. Because that’s ultimately what I think most parents are concerned about’ (Dan, Head of yr 8, S2)

#### Reporting symptoms to school

Most families and some staff anticipated under-reporting to school of student COVID-19 symptoms, due to embarrassment, wanting to attend school or parents needing to work. For staff symptoms, under-reporting reasons included a culture of presenteeism, and guilt at having exposed themselves to risk. Families wanted clarity about symptoms to report, method of reporting and implications (see box 2).

#### School COVID-19 testing

All staff and most parents thought testing in schools was important and would be enthusiastic about monthly testing. Testing at schools would reassure students, parents, and staff about school safety, and encourage attendance—a ‘massive selling point for schools’.

A minority of participants had concerns about school testing including:

- Parental concerns about use and anonymity of information, particularly among BAME families (see box 2).
- More school closures due to cases detected by testing, with implications for parents (time off work), students (loss of learning) and schools (attendance figures and academic achievement).
- Feasibility of testing the whole school—time, space and administration needed.

Staff suggested emphasising to families the importance of testing, including a potentially reduced risk of whole school closure (see box 2). They also emphasised the need for quick and minimally disruptive testing. Families wanted care, discretion and anonymity in notification of positive results.

## Discussion

Families and school staff had concerns about an increased COVID-19 risk with the full reopening of schools, particularly to vulnerable individuals, but on balance most felt the benefits outweighed the risks. Some staff anticipated guilt at their potential to spread COVID-19. Students, their parents and school staff generally felt planned school risk reduction measures were acceptable and pragmatic solutions to the perceived impossibility of social distancing between students in crowded schools. Negative unintended consequences of the new measures were anticipated on student behaviour, learning, pastoral care and particularly for those with SEN or mental health issues who may find the measures especially challenging. Stigma related to COVID-19 positivity was thought unlikely to be widespread in schools by staff, although families, particularly BAME families, anticipated possible stigma around COVID-19 diagnosis. Case reporting must, therefore, be managed sensitively. The imperative for testing in schools was recognised by staff and most parents, although with concerns over data security and feasibility.

Contrasting with previous data,^14^ students, parents and staff understood the need for risk reduction measures. Staff concerns about negative unintended consequences of the pandemic/risk reduction measures are shared by other UK teachers, policymakers and parents.^6 14 17 18^ Concerns have been raised particularly in relation to deprived communities and SEN students, with some evidence of a disproportionate effect on people with learning disabilities (in terms of COVID-19 deaths),^19^ worsening mental health among children^20^ especially those from BAME backgrounds,^21^ and worsening student behaviour.^22^ The impact on teachers appears similar to other front-line workers, with high anxiety, depression and distress levels, partly related to feelings of guilt at potentially infecting others and conflicting duties.^23 24^ In addition to previously noted feasibility concerns over risk reduction measures,^25 26^ we identified issues of year-group ‘bubble’ crossover and non-compliance due to forgetting and wanting to be ‘normal’/socialise and social distancing being impossible due to crowded school sites. The barriers we identified support calls for funding to help implement risk reduction measures (including hand-sanitiser, extra cleaning, free masks for all pupils and hiring extra teachers and teaching rooms so smaller class sizes can enable social distancing),^17 27^ with schools reportedly spending up to £8 k each on COVID-19 risk reduction measures in early reopening of campuses.^28^ BAME parents’ concerns about use of data for school COVID-19 testing echo scepticism and apprehension among BAME public contributors around COVID-19 vaccine trials.^29^

### Limitations

This study was conducted in a limited time frame in response to an urgent need to understand attitudes towards school COVID-19 mitigation measures and views on managing infections in schools. As a result, we took a pragmatic approach, capturing one time point in a rapidly changing field. Several limitations must be acknowledged. During the study, the community COVID-19 transmission rates in the research setting were relatively low; this context may have influenced participants risk perceptions. Furthermore, the sample lacks those with English as an additional language (recruitment materials were translated); those disengaged from school (recruitment was mainly via schools); and those without internet access/computers (due to COVID-19 restrictions we mainly used online recruitment). In addition, the majority of parents interviewed were mothers. Our findings have identified useful insights with important implications for schools, but should be interpreted with these limitations in mind.

### Implications

Due to lack of detailed school guidance, our interviews highlighted a wide variety of school COVID-19 mitigation measures, meaning comparison of effectiveness of mitigation measures will be difficult.

More could be done to reduce school transmission, such as reducing crowding to improve social distancing (eg, using other community spaces) and introducing effective school COVID-19 testing.^30–33^ These actions are even more vital as the pandemic progresses and family and staff opinions about the benefits of schools remaining open may shift. Even with the implementation of such measures, as demonstrated in the UK in early 2021, stopping face-to-face teaching may be a necessary last resort to reduce COVID-19 transmission.

Barriers to mitigation measure compliance may be addressed through clear, consistent information and reminders,^24 33^ and engendering a sense of collective responsibility.^34^ This applies both to communication from schools to families and from government to schools—our findings demonstrate that government have provided ambiguous recommendations rather than clear instructions which has left schools having to interpret them. Clear communication is needed from government based on latest scientific evidence. Our findings also demonstrate the importance of emphasising to students the collective good, benefits for their family or wider community, and a sense of collective identity and responsibility.^35–37^ This conflicts with UK government use of police and fines to enforce measures in wider society,^38^ for which behavioural scientists have expressed concern.^39 40^ Schools may need policies, rules and reward systems, and discipline^22 35^ but should consider a compassionate approach to COVID-19 mitigation measures, which seeks to promote collective identity and social norms, as well as being sensitive to potential stigma of COVID-19.^41^

Although our findings support schools returning to face-to-face teaching for all (risks from school closure outweigh those of COVID-19,^18^ policy-makers must consider potential unintended consequences of measures, and ways to help schools support vulnerable individuals and those with additional needs and avoid widening inequalities.^24^ This may include additional funding and resources to ensure that staff and pupils can adhere to the current government advice,^42^ and access to home learning resources for the most vulnerable. It may also involve training staff to meet the emotional and mental health needs of students^6^; for example, many organisations are suggesting a trauma informed approach for schools during this period.

### Future research

Further research is needed to understand the welfare and morale of staff and students as the pandemic continues to disrupt schools, including the mental and physical impact of self-isolation of groups of students, and the impact on learning and disruption to schools. Further research to identify the ongoing disproportionate impact of the COVID-19 pandemic, both within and outside schools, on vulnerable and deprived groups is vital. There is also a need for research to understand how to address BAME families’ particular concerns relating to COVID-19 transmission within schools.

## Conclusion

Families and staff support COVID-19 mitigation measures in schools as a means of students having face-to-face education. Clear messaging and engendering collective responsibility are important for compliance and success of mitigation measures. However, schools and policy-makers need to consider potential unintended consequences of measures, ways to support vulnerable individuals and those with additional needs, and avoidance of widening inequalities. Additional funding may be required.

## Supplementary Material

Author's manuscript

## Data Availability

Anonymised interview transcripts are available on reasonable request from University of Bristol Research Data Storage Facility. DOI for the data is 10.5523/bris.v5fc53z84q2q2wqd2omc8mdml
