# Supplementary material for: Reducing COVID-19 risk in schools: a qualitative examination of secondary school staff and family views and concerns in the South West of England
Source: BMJ Paediatr Open. 2021 Mar 10;5(1):e000987. doi: 10.1136/bmjpo-2020-000987 (PMC7948157; doi:10.1136/bmjpo-2020-000987)
Supplement: Supplementary data [file bmjpo-2020-000987supp001.pdf]

Supplementary material: Interview topic guides

School staff topic guide

1. **Demographic**

Check their role at school, how long they've been there /how long been teaching?

2. ***Experience of home learning***

Has your school been open during lockdown (for keyworker families) and what is the status now (which years are attending, approx how many of permitted children are attending etc). PROMPT: Years 8 and 10 in particular.

How have you been in contact with parents and pupils during lockdown? How much face to face teaching are you currently doing? How much are you physically in school?

What has been good / bad about teaching remotely during lockdown for you and the pupils?

What could be done/could have been done to improve the support for pupils (learning and in general) while they are at home?

Have you had feedback from parents and/or students about what they are finding difficult or what is working well?

Anything you would you like to see continue/lessons learnt that may help in the future? (PROMPTS: better communication with parents, online learning)

3. ***Attitude towards returning to school***

How do you feel about schools fully reopening in September? Why? Positives? Negatives? (PROMPTS: pupils can socialise with friends, structure to the day, educational attainment, wellbeing/mental health/anxiety, also positives and negatives for them being back at work) .

How do you feel about the government's guidance and suggested measures? Do you trust this guidance? Why do you feel this way? Is there anywhere you would prefer to get (trusted) information?

> Do you have any concerns about returning to school? Examples? (PROMPTS: Concern about risk of catching coronavirus, availability of PPE, worries about maintaining social distancing/bubbles, challenges of complying with government guidance, concern about personal or family members with underlying health issues, practicalities of timetabling lessons, behaviour of pupils, supporting pupils who have fallen behind, changes in teaching compared to during lockdown (less teacher attention, expectation stay at front of class etc))
>
> > Are you concerned about any particular student groups (or staff) and why? (pupils with SEN, pupils disengaged from school/struggled during lockdown, pupils from BAME communities, pupils/teachers/staff with family members who are vulnerable

to COVID-19, pupils without internet access, pupils on free school meals, particular year groups – years 8 and 10 in particular)

What is planned to support these pupils?

Are there particular groups that you feel are more cautious about sending their children back to school? How you trying to support these families?

What additional support would you like (local, national) to overcome barriers? (PROMPTS: support/information from school, availability of PPE; funding) . Is there anything that you have found helpful so far?

Have you heard from parents and/or students about their views on schools-re-opening? What are their concerns?

4. ***Attitude towards social distancing in schools***

What plans have your school put in place to facilitate social distancing? (PROMPTS: changing rooms, layout of school, changes to timetabling)

How easy / difficult do you think it will be/has been for pupils/school staff to follow social distancing in schools?

Do you have any concerns about the 'bubbles' idea and how it will work (e.g. staff crossing between bubbles)?

What could make it difficult for pupils/school staff to do this? (e.g. dedicated staff/time to do the extra work, built environment/classroom space, children remembering, fighting/bullying) Are there any particular groups of pupils that find it easier/more difficult? (older children, SEN, cultural issues, children who have been at school throughout).

What could help pupils do this? (additional classroom space / teachers, markers on the ground, verbal and written information, emphasis on reducing illness generally, reward/sanction system)

What times in the day will it be easier/more difficult for pupils to social distance from others? (PROMPTS: journey to/from school, arriving/leaving school, break and lunch times, during lessons, moving between lessons, PE lessons, in bathrooms,)

Any thoughts on whether pupils are/will be social distancing outside school?

5. ***Attitude towards hand hygiene and infection control strategies in schools***

What policies have been put in place to encourage increased of frequency and thoroughness of handwashing by students? How about touching of the face? Ventilation (SAGE advice hourly)? How about cleaning in school? Any other infection control measures? Any guidance on masks?

So far what has been the students' attitude to handwashing ?

Impact of new handwashing & hygiene practices on time dedicated to learning?

How easy / difficult is it and do you think it will be for pupils/school staff to wash hands more regularly in schools?

What could stop pupils/school staff doing this? (already doing as much as they can, availability of hand washing facilities, bullying in bathrooms)

What has helped and could help pupils/school staff do this? (behaviour change intervention, verbal and written reminders, handwashing incorporated into timetabling, additional facilities, provision of hand sanitisers)

What times in the day will it be easier/more difficult to do this?

6. Impact on learning

Do you have any comments on how you think lockdown/school closure/social distancing in schools has/will affect pupil's learning? How will your teaching methods need to change?

What could help to improve this/reduce any negative impact? Guidance, funding, etc?

7. ***Plans for infections***

Does the school have a plan for dealing with any suspected/diagnosed Covid infections? (keeping kids off school, reporting mechanisms, testing, contact tracing, school closure)? Will you increase risk reduction measures (in line with recent SAGE advice) e.g. masks, smaller bubbles, no group working?

Would there be any issues if a class or year group needed to self-isolate? How would you feel about having to self-isolate (for at least 7 days) if pupils /you/other staff developed the symptoms of coronavirus? Negatives? Positives?

Is it possible for you to self-isolate?
What could stop you self-isolating?
What could help you self-isolate?

What is the biggest issue if a whole school local lockdown is required, with pupils to remain at home? Why? If school lockdown happens, school will have to provide remote education support – what would the school provide and would there be any challenges?

8. ***acceptability of possible new school test-and-trace study to map Covid-19 infections in students and staff using saliva sampling***

Researchers at the University of Bristol are developing a possible new study. This study will help understand how the virus spreads among young people and in schools. The study will involve testing young people in certain school years *and school staff for coronavirus using saliva sampling system (taken yourself) once a month for 6 months. It would also require reporting of symptoms to the school. Infection will be linked to home postcode and linked to NHS data to understand who is getting*

the virus and where. A system will be developed and tested that uses different sources of information to enable contact tracing and help control outbreaks.

How would you feel about a mapping study like this in your school? Positives? Negatives? Is testing once a month feasible? Test will be spitting into a tube, would there be any problems you all doing this together in public in your tutor group? – why? How overcome?

Anything that would make it difficult or easier? What would be an incentive for your school to take part?

Are there any issues specific to years 2,5,8,10,12?
Any suggestions on how we should present it to young people/families?
How to alert individuals, classes, school of suspected cases?
How best to provide tests? When? In classrooms, take home kits, school nurses office?
How to take consent? Student consent ?

How would you feel if you tested positive? Or if someone in the school did? Stigma if pupils/school staff develop symptoms, if outbreak ripples into community, concern for health

If there was a mapping study like this one in your school and you developed symptoms, how likely is it that you would report them? Same question for pupils/other staff?

> What would be the most important reason you /pupils/other staff would decide to report symptoms of coronavirus?
> What would be the most important reason that would stop pupils/you/other staff from reporting symptoms of coronavirus? (PROMPTS: stigma, bullying, data protection concerns)

The study would also provide an intervention to help reduce the risks of Covid, which may be a modified version of Germ Defence (Germ Defence is a website that provides advice on infection control measures and helps users think about when and how to carry out key infection control behaviours such as handwashing and cleaning, avoiding sharing rooms and surfaces, managing incoming deliveries, and ventilating rooms. Behaviour change techniques are used to help people implement this advice).

What might be useful for school to help them implement new measures/interventions like this?

Parent/Guardian topic guide

1. ***Background question***

How old is your secondary school aged child(ren)?
What area of Bristol do you live in?
[may need to ask this at the end without child] Do you have any particular concerns about Covid-19?

2. ***Experience of home learning – don't spend too long on this section***

Has your child(ren) been into school during the lockdown? **N.B. Tailor subsequent questions depending on whether child has been into school.**

What has been good / bad about your son/daughter being out of school during lockdown? Has there been an emotional impact of social distancing/isolation/not attending school?

What could be done to improve your / your son/daughter's experience of home learning or other things they are missing from school?

Anything you would like to see continue?

What is the biggest issue if a whole school local lockdown is required, with pupils to remain at home? Why? If school lockdown happens, school will have to provide remote education support - when teaching pupils remotely, what would you expect the schools to provide to support home learning as a minimum ? Why ?

3. ***Attitude towards returning to school***

How do you feel about your son/daughter returning to school in September? Positives? Negatives? (PROMPTS: support parents to work, young person can socialise with friends, structure to the day, educational attainment, wellbeing)

Where has your information about school reopening come from - school, council, government, other parents? Which information would you trust the most when thinking about going back to school and making decisions around this?

Do you have any concerns about your son/daughter returning to school? Examples? (PROMPTS: Concern about risk of catching coronavirus, worries about maintaining social distancing, concern about family members with underlying health issues, pressures of work/catching up). Is there anything particular about their school year/age?

What would like the school to do to help address your concerns? (PROMPTS: support/information from school)

What additional support would you like(local, national) to overcome concerns ?

4. ***Attitude towards social distancing in schools***

How has your child(ren) found social distancing so far?

Do you think the school need to do things like social distancing and hand washing? Why/why not?

Do you know what plans the school has to enable social distancing in schools? (PROMPTS: changing rooms, layout of school, limiting number of children in school, a mix of online and face to face teaching)

The government has also brought in the idea of year bubbles, what are your views on that? Do you think it will work? Will it work outside of school?

How easy / difficult do you think it will be for young people to follow social distancing in schools?

> What could stop young people from doing this? (e.g. built environment/classroom space, fighting/bullying, peer pressure)
>
> What could help young people do this? (additional classroom space / teachers, markers on the ground, verbal and written information)
>
> What times in the day will it be easier/more difficult for young people to social distance from others? (PROMPTS: commute to school, arriving/leaving school, break and lunch times, during lessons, moving between lessons, PE lessons, in bathrooms, after school, weekends)

Do you think social distancing measures will affect learning?

5. ***Attitude towards hand hygiene in schools***

How has your child(ren) found following hand washing advice so far? Do you have any concerns about possible negative impact of extra hand-washing e.g. compulsive behaviour or less exposure to germs? Anything that could be done to address these concerns?

How important do you think hand washing is at school (re Corona)? Do you know what the school is putting in place to encourage frequency and thoroughness of handwashing by students ?

How easy / difficult do you think it will be for young people to wash hands in schools?

> What could stop young people doing this? (availability of hand washing facilities, bullying in bathrooms)
>
> What could help young people do this? (verbal and written reminders, handwashing incorporated into timetabling, additional facilities, provision of hand sanitisers)
>
> What times in the day will it be easier/more difficult to do this?

6. ***Acceptability of test-and-trace study***

Researchers at University of Bristol are developing a possible new school test-and-trace study to map Covid-19 infections in students and staff This study will help understand how the virus spreads among young people and in schools. The study will involve testing young people and school staff for coronavirus using saliva sampling (taken yourself) once a month for 6 months, and reporting symptoms to school.. infection will be linked to home postcode & linked to NHS data to understand who is getting the virus & where. A system will be developed and tested that uses different sources of information to enable contact tracing and help control outbreaks.

How would you feel about a mapping study like this in your child's school? Positives? Negatives?

Only positive test results would be sent out (no news = good news) – would you have any concerns about this? Also positive test results will be sent to Headteacher, Bristol City Council Health Protection Team & nationally Public Health England – do you have any issues with this? Why?

Test will be spitting into a tube, would there be any problems everyone doing this together in public in your tutor group? – why? How overcome? If there is a confirmed case from school testing – all members of household/contacts will be asked to provide weekly samples for 4 weeks – how would you feel about this? Any problems with this? Why? How to do this without causing issues/concerns

Would you have any concerns about your child taking part in a test and trace system like this? What would help allay those concerns?

What age do you think it would be appropriate for children to give their own consent for this? How would you feel about your child(ren) giving their own consent? Why?

If there was a mapping study like this, how likely do you think it would be that children at school would report symptoms of coronavirus? What would be the barriers/facilitators?

How would you feel about your family having to self-isolate (for at least 7 days) if you/your child developed the symptoms of coronavirus? Negatives? Positives?

Do you have the space at home for your child to self isolate?

How would you feel about the whole family having to self-isolate if someone you/your child had been in contact with developed the symptoms of coronavirus?

Young person's topic guide

1. ***Experience of lockdown and education– don't spend too long on this section***

Have you been into school during the lockdown? **N.B. Tailor subsequent questions depending on whether they have been into school.**

What has been good / bad about home learning (e.g. learning remotely) during lockdown? What have you missed/not missed about school? How have you been feeling during this time?

What has helped during this time?

What could have the school done to help make it easier?

How do you think home learning has affected your learning?

PROMPTS : What could be done to improve experience of home learning? What would you like to keep doing?

2. ***Attitude towards returning to school***

How do you feel about returning to school in September? Positives? Negatives? (PROMPTS: socialising with friends, structure to the day, education, wellbeing, concern about risk of catching coronavirus, worries about maintaining social distancing)

Where has your information about school reopening come from - school, council, government, other parents? Which information would you trust the most when thinking about going back to school and making decisions around this?

Do you have any concerns or worries about returning to school? Examples? (PROMPTS: Concern about risk of catching coronavirus, worries about maintaining social distancing, concern about family members with underlying health issues, peer relationships)

What would help you feel better about these worries or what would help solve those worries? (PROMPTS: support/information from school)

Do you think your learning will be the same?

Are you aware of the school's plans for future lockdown/2nd wave/local lockdown? How do you feel about these plans e.g. a mix of online and face to face teaching?

3. ***Attitude towards social distancing in schools***

How have you found social distancing so far (keeping 2m apart from people)? How much do you think people your age have followed social distancing when they are out?

What has made it difficult to social distance? What could make it easier?

Do you think the school need to do things like social distancing and hand washing? Why/why not?

How easy / difficult do you think it will be to follow social distancing (stay 2 metres apart from others) in schools?

To help with social distancing, the government has also brought in the idea of year bubbles, where you are asked to stay in your class/year group and not mix with other outside it, what are your views on that? Do you think it will work? Will it also work outside of school?

What could stop you from doing this? (e.g. built environment/classroom space, fighting/bullying/wanting to feel close to friends again)

What could help you do this? (additional classroom space / teachers, markers on the ground, verbal and written information) do you know what the school is planning to make it easier for you?

What times in the day will it be easier/more difficult to social distance from others? (PROMPTS: journey to/from school, arriving/leaving school, break and lunch times, during lessons, moving between lessons, PE lessons, in bathrooms, after school and at weekends)

Do you think the changes at school will affect your learning?

4. ***Attitude towards hand hygiene in schools***

What have you been told so far about washing your hands? How important do you think hand washing is at school (re Corona)?

Do you know what your school is putting in place to encourage and handwashing ?
How easy / difficult do you think it will be to wash hands more regularly in schools?

What could stop you doing this? (availability of hand washing facilities, bullying in bathrooms)

What could help you do this? (verbal and written reminders, handwashing incorporated into timetabling, additional facilities, provision of hand sanitisers)

What times in the day will it be easier/more difficult to do this?

5. ***Acceptability of test-and-trace study***

Ascertain if they know much about the national test and trace scheme and tailor this description accordingly. Researchers at the University of Bristol are developing ways to test young people and school staff for coronavirus using saliva samples (you spit into a container). This study will help them understand how many people have the virus and how it gets passed on. Where the virus is found it will be linked to the person's home postcode and linked to a NHS database to help see which people have the virus and where they live. The researchers would also look at ways to trace who that person had been mixing with. This information may help control the virus and stop outbreaks.

How would you feel about a test and trace study (like the one I've just described) in your school? Positives? Negatives? Is testing once a month feasible?

What would be the best way to take saliva samples in school? In tutor groups, classrooms, take home kits, school nurses office? Test will be spitting into a tube, would there be any problems you all doing this together in public in your tutor group? – why? How overcome?

Only positive test results would be sent out (no news = good news) – would you have any concerns about this? What would be the best way to be told if you tested positive? Also positive test results

will be sent to Headteacher, Bristol City Council Health Protection Team & nationally Public Health England – do you have any issues with this? Why?

If there is a confirmed case from school testing, all members of household/contacts will be asked to provide weekly samples for 4 weeks – how would you feel about this? Any problems with this? Why? How to do this without causing issues/concerns?

Do you know anyone who has had a positive Corona test? (if not then ask hypothetically if they/someone else did test positive). Do you think that person minded/would mind other people knowing or were/might be treated unkindly by anyone or how they felt /might feel about it? Why? PROMPTS: Stigma, concern about health, concern about having to stay home again

How would you feel about people tracing who you had been mixing with?

If there was a test and trace study like this one in your school and you developed coronavirus symptoms, how likely is it that you would report them?

Why would you decide to report symptoms of coronavirus?

Why might you decide not to report symptoms of coronavirus? (PROMPTS: stigma, bullying, data protection concerns, make sure cover concerns about testing and also concerns about being diagnosed)

How would you feel about having to self-isolate (for at least 7 days) if you developed the symptoms of coronavirus or tested positive? Negatives? Positives?

Do you have the space at home to self isolate?

What could stop you self-isolating?

What could help you self-isolate?

How would you feel about having to self-isolate if someone you had been in contact with developed the symptoms of coronavirus?
