# Supplementary material for: Reducing COVID-19 risk in schools: a qualitative examination of secondary school staff and family views and concerns in the South West of England
Source: BMJ Paediatr Open. 2021 Mar 10;5(1):e000987. doi: 10.1136/bmjpo-2020-000987 (PMC7948157; doi:10.1136/bmjpo-2020-000987)
Supplement: Author's manuscript [file bmjpo-2020-000987.draft_revisions.pdf]

BMJ Paediatrics Open

BMJ Paediatrics Open is committed to open peer review. As part of this commitment we make the peer review history of every article we publish publicly available.

When an article is published we post the peer reviewers' comments and the authors' responses online. We also post the versions of the paper that were used during peer review. These are the versions that the peer review comments apply to.

The versions of the paper that follow are the versions that were submitted during the peer review process. They are not the versions of record or the final published versions. They should not be cited or distributed as the published version of this manuscript.

BMJ Paediatrics Open is an open access journal and the full, final, typeset and author-corrected version of record of the manuscript is available on our site with no access controls, subscription charges or pay-per-view fees (http://bmjpaedsopen.bmj.com).

If you have any questions on BMJ Paediatrics Open's open peer review process please email info.bmjpo@bmj.com

BMJ Paediatrics Open

Reducing Covid-19 risk in schools: a qualitative examination of secondary school staff and family views and concerns

Journal:	*BMJ Paediatrics Open*
Manuscript ID	bmjpo-2020-000987
Article Type:	Original research
Date Submitted by the Author:	14-Dec-2020
Complete List of Authors:	Lorenc, Ava; University of Bristol,<br>Kesten, Joanna; University of Bristol School of Social and Community Medicine, NIHR Collaboration for Leadership in Applied Health Research and Care West and NIHR Health Protection Research Unit in Evaluation of Interventions<br>Kidger, Judi; University of Bristol<br>Langford, Rebecca; University of Bristol<br>Horwood, Jeremy; University of Bristol
Keywords:	COVID-19

SCHOLARONE™
Manuscripts

I, the Submitting Author has the right to grant and does grant on behalf of all authors of the Work (as defined in the below author licence), an exclusive licence and/or a non-exclusive licence for contributions from authors who are: i) UK Crown employees; ii) where BMJ has agreed a CC-BY licence shall apply, and/or iii) in accordance with the terms applicable for US Federal Government officers or employees acting as part of their official duties; on a worldwide, perpetual, irrevocable, royalty-free basis to BMJ Publishing Group Ltd ("BMJ") its licensees and where the relevant Journal is co-owned by BMJ to the co-owners of the Journal, to publish the Work in this journal and any other BMJ products and to exploit all rights, as set out in our licence.

The Submitting Author accepts and understands that any supply made under these terms is made by BMJ to the Submitting Author unless you are acting as an employee on behalf of your employer or a postgraduate student of an affiliated institution which is paying any applicable article publishing charge ("APC") for Open Access articles. Where the Submitting Author wishes to make the Work available on an Open Access basis (and intends to pay the relevant APC), the terms of reuse of such Open Access shall be governed by a Creative Commons licence – details of these licences and which Creative Commons licence will apply to this Work are set out in our licence referred to above.

Other than as permitted in any relevant BMJ Author's Self Archiving Policies, I confirm this Work has not been accepted for publication elsewhere, is not being considered for publication elsewhere and does not duplicate material already published. I confirm all authors consent to publication of this Work and authorise the granting of this licence.

Confidential: For Review Only

Reducing Covid-19 risk in schools: a qualitative examination of secondary school staff and family views and concerns

Dr Ava Lorenc, Senior Research Associate[1, 2]

Dr Joanna M Kesten, Senior Research Associate [1, 2, 3]

Dr Judi Kidger, Lecturer[2]

Dr Rebecca Langford, Research Fellow[2]

Dr Jeremy Horwood [1, 2, 3] (corresponding author; j.horwood@bristol.ac.uk)

1. National Institute for Health Research Applied Research Collaboration West (NIHR ARC West), University Hospitals Bristol and Weston NHS Foundation Trust, 9th Floor, Whitefriars, Lewins Mead, Bristol, BS1 2NT, UK.
2. Population Health Sciences, Bristol Medical School, University of Bristol, Canynge Hall, 39 Whatley Road, Bristol, BS8 2PS, UK
3. NIHR Health Protection Research Unit (HPRU) in Behavioural Science and Evaluation at University of Bristol Canynge Hall, 39 Whatley Road, Bristol, BS8 2PS, UK

Confidential: For Review Only

Abstract

Objective To investigate student, parent/carer, and secondary school staff attitudes towards school Covid-19 mitigation measures.

Methods Recruitment used school communication, community organisations and snowball sampling. Audio recorded online or phone individual/group semi-structured interviews were conducted in July-Sept 2020 and lasted 45 minutes. Interviews focused on views towards social distancing, hand-hygiene and testing. Framework analysis of interview notes and transcripts.

Results

Participants were 13 school staff, 20 parents and 17 students (11-16 years). Concerns about Covid-19 risk at school, especially to vulnerable individuals, were outweighed by perceived risks of students missing out on learning. Some staff felt guilt around being a potential “spreader” by teaching multiple classes. Findings highlighted a wide variety of school covid-19 mitigation measures being deployed due to ambiguous government guidance. Participants generally saw mitigation measures as an acceptable and pragmatic solution to the perceived impossibility of social distancing in crowded schools, although anticipated challenges in changing habitual behaviour. Participants supported school Covid-19 testing but identified the need to consider data security and stigma around Covid-19 diagnosis. Staff were concerned about unintended consequences of risk-reduction strategies on student behaviour, learning, and pastoral care, particularly for those with Special Educational Needs or mental health issues who may find the measures especially challenging, and resultant widening inequalities.

Conclusion

Families and staff supported Covid-19 mitigation measures in schools and would welcome the roll out of a school test-and-trace programme. Clear messaging and engendering collective responsibility are important for compliance and success of Covid-19 mitigation measures. However, schools and policymakers should consider unintended consequences of measures, providing extra support for vulnerable students and those with additional needs, and consider ways to avoid widening educational and health inequalities. Findings demonstrate the feasibility of effectively implementing school Covid-19 infection control measures should be influenced by the balance of risks and benefits to student.

Confidential: For Review Only

What is known about the subject

- Schools reopened in September 2020 with Covid-19 risk reduction measures in place. Compliance with and impact of these measures – especially on vulnerable groups - is unclear.
- Current UK government Covid-19 policy includes punitive measures to ensure behavioural compliance.
- Scientists have advised scaling-up testing and tracing within schools

What this study adds

- Given the challenges of social distancing under current government school guidelines, families and staff view effective test-and-trace and innovative risk reduction measures as imperative
- Schools should adopt an encouraging and compassionate approach to implementing risk reduction measures, with clear messaging emphasising collective responsibility.
- Families and staff support schools remaining open but concerns over unintended consequences of risk reduction measures must be addressed by schools and policy makers.

Introduction

To reduce the spread of Covid-19, a UK 'lockdown' was announced on 23rd March 2020 and school campuses were closed to all but vulnerable or priority students. In July, the government announced all school campuses would fully reopen in September.

Evidence for school closures reducing infection spread is equivocal [1]. Those in favour of school campuses reopening to all pointed to the impact on learning [2], particularly for lower socioeconomic groups [3], widening inequalities and consequences for students' physical and mental health [4-10]. Remote learning also affected staff wellbeing and mental health [11]. However, schools reopening carried risks - modelling suggested increased Covid-19 infection would occur if schools reopened full time (combined with easing of other restrictions and without scaled up test-and-trace systems) [12].

Government guidance set out a range of school-based protective measures to reduce Covid-19 outbreaks, including hand-hygiene, cleaning, reporting symptoms, and social distancing. Further, a UK modelling study recommended existing testing should be scaled up[12 13].

Understanding the views and concerns of school staff, parents and students about the implementation of this guidance is key to ensuring compliance, avoiding unintended harms[13] and supporting schools to reduce risks. This secondary school qualitative study aimed to rapidly explore student, parent/carer and school staff attitudes towards school Covid-19 mitigation measures, views on managing Covid-19 infections in schools and opinions about student groups who may be particularly affected by these measures.

ntial: For Review Only

Methods

Study Setting

Between July and September 2020, we recruited 11-16 year olds, parents and school staff through secondary schools and local community organisations in the South West of England.

Sampling and recruitment

Because lower socioeconomic and Black, Asian and Minority Ethnic (BAME) populations are disproportionately affected by Covid-19, we contacted 21 schools with higher levels of these populations to ensure we captured their specific concerns. Participating schools sent study information to potential participants. We focused student/parent recruitment on Year 8 (age 12-13; most had been out of school since March) and Year 10 (age 14-15; faced key exams next year). We also advertised the study via community organisations in areas of higher BAME groups/deprivation (newsletters, social media, and direct contact with members) and used snowball sampling. Individuals interested in participating contacted the researcher directly to arrange an interview. All eligible volunteers were interviewed (none refused or dropped out).

Interviews

Participants chose to be interviewed on their own, with a friend and/or parent (students) or with colleagues (staff). AL led interviews by phone or video, with JMK or JK present for group interviews/student interviews without a parent present. Interviews were 45 minutes and audio recorded. Participants provided audio-recorded verbal informed consent (or assent and parent consent for those under 16 years).

Students and school staff provided input into the study design and topic guides.

Topic guides covering attitudes towards social distancing, school hand-hygiene and infection control strategies, and acceptability of a test-and-trace system were used flexibly, allowing exploration of issues raised by participants. We gave participants a shopping voucher as thanks for their time.

Data collection was informed by the concept of information power [14]and pragmatic considerations of the project timeline.

Patient and public involvement

The NIHR ARC West Young Peoples Advisory Group provided feedback on study design and interview topic guides.

Analysis

Confidential: For Review Only

95 Producing timely reports for local and national stakeholders necessitated rapid analysis (reports
96 available at https://arc-w.nihr.ac.uk/research/projects/the-back-to-school-study). AL used the
97 framework method[15] to analyse anonymised notes taken during interviews, and re-listening to
98 aspects of the interviews. Notes were coded deductively using topic guide headings to develop a
99 coding framework in Microsoft EXCEL and identify themes. Framework analysis was appropriate for
100 the specific a priori questions and limited time frame.

101 Audio recordings were then transcribed and anonymised. We analysed transcripts collaboratively,
102 reading them through and adding new subthemes and quotes to the existing framework.

103 The study was approved by the Faculty of Health Sciences Committee for Research Ethics, University
104 of Bristol (ref 108084).

Confidential: For Review Only

Results

Participants

Participants were 13 school staff (Heads/Assistant Heads, teachers, Special Educational Needs Coordinators (SENCOs)) from seven schools, and 20 families from ten schools (12 with a BAME child/parent) - 17 students (mostly years 7/8/10; 9 females; 8 males) and 20 parents (19 mothers, 1 father) (Table 1).

Covid-19 risk concerns

Most staff and around half of families (students and parents) were not concerned about personal/family Covid-19 risk from returning to school (Table 2). However, many staff anticipated increased cases describing schools as "petri dishes". Some staff worried about being a potential "spreader" (Table 2). Staff also had concerns about higher risk staff and students, including those from BAME backgrounds, those who were pregnant or had health conditions.

Many families were more concerned about the negative educational consequences of students not being at school, accepting that home schooling cannot continue indefinitely. However, families did note the risk to vulnerable family members (due to health or age) (Table 2). Concern was more common in families with BAME members, although a minority explicitly cited ethnicity-related risk, with some noting the lack of scientific understanding around this risk.

School risk reduction measures

Outside school, most students had accepted social distancing, wearing masks and handwashing, understanding their necessity, with minor negative comments such as social distancing being "not nice". Most aimed to social distance and a few had socialised little and enjoyed staying at home during the first lockdown.

Interviewees highlighted a wide variety of school Covid-19 risk reduction measures (see Box 1) . Participants agreed such measures were needed, although staff were frustrated with the lack of detail in government guidance, meaning each school had to develop their own plans. Staff were generally happy with schools' plans but had reservations about feasibility and how they would "play out in practice".

Confidential: For Review Only

Barriers/concerns

All participants agreed student social distancing was impossible and "pointless" given the numbers of people and lack of space (Table 3). Year 'bubbles' (see Box 1) were seen as a "pragmatic solution", although there was concern about crossover via siblings or groups leaving school together, and teachers (though less commonly reported). In terms of compliance with risk reduction measures, of most concern among families was forgetting, and a desire to "be normal".

Staff and family concerns about hand-hygiene/infection control were mostly practical, including: availability of resources (sanitiser/soap, sinks, cleaners) - one school estimated a £40k cost of hand sanitiser; bathroom cleanliness; time for handwashing; and effective use of measures e.g. hand sanitiser vs. washing, or proper use of masks. Some were concerned about ventilation e.g. windows not opening.

A minority of staff were worried about behavioural issues arising from students having to stay in the same classroom e.g. unsupervised lesson changeovers, student boredom and lack of movement. Other concerns included risk of using public transport and reduced range of lessons/activities. An important concern for staff was the impact of these measures on learning and pastoral care, especially social distancing measures, e.g. fewer interactive lessons, and less opportunity to support individual students.

Staff were concerned social distancing measures would particularly affect students with special educational needs (SEN) or mental health issues. Particular issues for students with SEN included (Table 3): struggling to understand and comply with the changes; finding less interactive lessons challenging; SEN staff being unable to work physically closely with students; physical needs e.g. personal care or feeding; and removal of 'safe spaces' (due to bubbles and infection risk from soft furnishing).

Facilitators/suggestions

The main suggestion from staff for facilitating the new rules was educating students about their importance, and encouraging a "we culture" of collective responsibility through a supportive, considerate approach (Table 3), although a minority of families thought handwashing should be compulsory and enforced.

Confidential: For Review Only

Staff and parents suggested clear consistent messaging and daily reminders, both verbal and visual. Clear and regular communication from schools about the measures would also reassure parents (Table 3).

Other suggestions to support the mitigation measures included:

- Funding for cleaning products, PPE and hand-sanitiser; additional classroom equipment to ensure no sharing
- Government guidance on PPE and SEN
- Staff training on how to work within the new measures
- Risk assessments for vulnerable students/staff
- Shorter lessons to allow time for handwashing
- Students bringing their own hand-sanitiser
- Encouraging staff to stay home if displaying symptoms (a culture shift from presenteeism common in schools) (Table 3).

School management of Covid-19 cases

Reaction to Covid-19 cases

Several families anticipated possible stigma around Covid-19 diagnosis. Staff did not generally anticipate stigma, due to increasing understanding of Covid-19 among students , and school communities having a generally tolerant and accepting attitude due to existing school population diversity - although two mentioned possible "mass hysteria" and another that teenagers "love to joke and point a finger" (see Table 4).

Reporting symptoms

Most families and some staff anticipated under-reporting of student Covid-19 symptoms, due to embarrassment, wanting to attend school, or parents needing to work. For staff symptoms, underreporting reasons included a culture of presenteeism, and guilt at having exposed themselves to risk. Families wanted clarity about symptoms to report, method of reporting and implications (see Table 4).

Test-and-trace

All staff and most parents thought testing in schools was important and would be enthusiastic about monthly testing. Testing at schools would reassure students, parents, and staff about school safety, and encourage attendance - a "massive selling point for schools".

A minority of participants had concerns about a school test-and-trace system, including:

Confidential: For Review Only

- Parental concerns about use and anonymity of information, particularly among BAME families (see Table 4).
- More school closures due to cases detected by testing, with implications for parents (time off work), students (loss of learning) and schools (attendance figures and academic achievement).
- Feasibility of testing the whole school – time, space and administration needed

Staff suggested emphasising to families the importance of testing, including a potentially reduced risk of whole school closure (see Table 4). They also emphasised the need for quick and minimally disruptive testing. Families wanted care, discretion and anonymity in notification of positive results.

Confidential: For Review Only

Discussion

Families and school staff had concerns about an increased Covid-19 risk with the reopening of schools, particularly to vulnerable individuals, but on balance most felt the benefits outweighed the risks. Some staff anticipated guilt at their potential to spread Covid-19. Students, their parents and school staff generally felt planned school risk reduction measures were acceptable and pragmatic solutions to the perceived impossibility of social distancing between students in crowded schools. Negative unintended consequences of the new measures were anticipated on student behaviour, learning, pastoral care, and particularly for those with SEN or mental health issues who may find the measures especially challenging. Stigma related to Covid-19 positivity was thought unlikely to be widespread in schools but case reporting must be managed sensitively. The imperative for testing in schools was recognised by staff and most parents, albeit with concerns over data security and feasibility.

Contrasting with previous data [16], students, parents and staff understood the need for risk reduction measures. Staff concerns about negative unintended consequences of the pandemic/risk reduction measures are shared by other UK teachers, policymakers, and parents[5 16-18]. Concerns have been raised particularly in relation to deprived communities and SEN students, with some evidence of a disproportionate effect on people with learning disabilities (in terms of Covid-19 deaths)[19], worsening mental health among children[20] especially those from BAME backgrounds[21], and worsening student behaviour[22]. The impact on teachers appears similar to other frontline workers, with high anxiety, depression and distress levels, partly related to feelings of guilt at potentially infecting others and conflicting duties[23]. In addition to previously noted feasibility concerns over risk reduction measures [24 25], we identified issues of year-group 'bubble' crossover and non-compliance due forgetting and wanting to be 'normal'/socialise. Our data support calls for funding to help implement risk reduction measures (including hand sanitizer, extra staff to cover absences and hiring extra buildings to enable social distancing) [17 26], with schools reportedly spending up to £8k each on Covid-19 risk reduction measures in early reopening of campuses [27]. BAME parents' concerns about use of data for test-and-trace echo scepticism and apprehension among BAME public contributors around Covid-19 vaccine trials [28].

Limitations

Lack of participants possibly most adversely affected, including: those with English as an additional language (recruitment materials were translated); those disengaged from school (recruitment was

Confidential: For Review Only

mainly via schools); and those without internet access/computers as, (due to Covid-19 restrictions we mainly used online recruitment). Also 19/20 parents were mothers.

Implications

Due to lack of detailed school guidance, our interviews highlighted a wide variety of school covid-19 mitigation measures, meaning comparison of effectiveness of mitigation measures will be difficult.

More could be done to reduce school transmission, such as reducing crowding to improve social distancing. (e.g. using other community spaces) and introducing an effective school test-and-trace system[29-31] These actions are even more vital as the pandemic progresses and family and staff opinions about the benefits of schools remaining open may shift.

Barriers to mitigation measure compliance may be addressed through clear, consistent information and reminders, and engendering a sense of collective responsibility [32 33]. Our findings demonstrate the importance of emphasising to students the collective good, benefits for their family or wider community, and a sense of collective identity and responsibility[34-36]. This conflicts with UK government use of police and fines to enforce measures wider society [37], for which behavioural scientists have expressed concern [38 39]. Schools may need policies, rules and reward systems, and discipline [22 34] but should consider a compassionate approach to Covid-19 mitigation measures, which seeks to promote collective identity and social norms, as well as being sensitive to potential stigma of Covid-19[40].

Although our findings support schools staying open (risks from school closure outweigh those of Covid-19)[18], policymakers must consider potential unintended consequences of measures, and ways to help schools support vulnerable individuals and those with additional needs and avoid widening inequalities. This may include additional funding to ensure adequate staff support and access to home learning resources for the most vulnerable. It may also involve training to meet the emotional and mental health needs of students[5] ; for example many organisations are suggesting a trauma informed approach for schools during this period.

Future research

Further research is needed to understand the welfare and morale of staff and students as the pandemic continues to disrupt schools. Particularly to understand the impact of groups of students having to self-isolate on their mental and physical health, and learning and disruption to schools. Further research to identify the ongoing disproportionate impact of the Covid-19 pandemic, both within and outside schools, on vulnerable and deprived groups is vital. There is also a need for

Confidential: For Review Only

research to understand how to address BAME families' particular concerns relating to Covid-19 transmission within schools.

Conclusion

Families and staff support Covid-19 mitigation measures in schools as a means of students having face-to-face education. Clear messaging and engendering collective responsibility are important for compliance and success of mitigation measures. However, schools and policymakers need to consider potential unintended consequences of measures, ways to support vulnerable individuals and those with additional needs, and avoidance of widening inequalities.

Funding

This work was supported by National Institute for Health Research, Applied Research Collaboration West (NIHR ARC West) and NIHR Health Protection Research Unit in Behavioural Science and Evaluation (NIHR HPRU BSE), grant number P520. JK's time was supported by NIHR School for Public Health Research. The views expressed are those of the authors and not necessarily those of the NIHR, the Department of Health and Social Care.

Competing interests

No authors have any conflict of interest to declare.

Author contributions

All authors have been involved in drafting the paper, read and approved the final version. AL recruited participants, collected and analysed data and led the writing of this manuscript. JK and JMK assisted with data collection. All authors collaborated on analysis.

Data sharing

Interview transcripts are available on request from University of Bristol Research Data Storage Facility.

Acknowledgements

We would like to thank the valuable contribution of our participants and the community groups who helped with recruitment: Baggator; Barton Hill Activity Club; Black South West Network; Bristol Somali Voice; Bristol Somali Forum; BS5 Secondary Education Forum; The Care Forum; Felix Road Adventure Playground; Off The Record; St George Community Centre; Ujima Radio; Up Our Street;

Confidential: For Review Only

Young Bristol. We are also grateful to the ARC West YPAG and teachers who were involved in designing the study.

Confidential: For Review Only

References

1. Viner RM, Russell SJ, Croker H, et al. School closure and management practices during coronavirus outbreaks including COVID-19: a rapid systematic review. *The Lancet Child & Adolescent Health* 2020;4(5):397-404. doi: https://doi.org/10.1016/S2352-4642(20)30095-X
2. Garbe A, Ogurlu U, Logan N, et al. COVID-19 and Remote Learning: Experiences of Parents with Children during the Pandemic. *American Journal of Qualitative Research* 2020;4(3):45-65.
3. Pensiero N, Kelly A, Bokhove C. Learning inequalities during the Covid-19 pandemic: how families cope with home-schooling 2020 [Available from: https://www.southampton.ac.uk/publicpolicy/covid19/learning-inequalities-covid-19.page.
4. Armitage R, Nellums LB. Considering inequalities in the school closure response to COVID-19. *The Lancet Global Health* 2020;8(5):e644. doi: 10.1016/S2214-109X(20)30116-9
5. Lundie D, Law J. Teachers' Responses and Expectations in the COVID-19 School Shutdown Period in the UK: University of Glasgow; 2020 [Available from: http://eprints.gla.ac.uk/221329/.
6. Patrick SW, Henkhaus LE, Zickafoose JS, et al. Well-being of Parents and Children During the COVID-19 Pandemic: A National Survey. *Pediatrics* 2020:e2020016824. doi: 10.1542/peds.2020-016824
7. Van Lancker W, Parolin Z. COVID-19, school closures, and child poverty: a social crisis in the making. *The Lancet Public Health* 2020;5(5):e243-e44. doi: 10.1016/S2468-2667(20)30084-0
8. Crawley E, Loades M, Feder G, et al. Wider collateral damage to children in the UK because of the social distancing measures designed to reduce the impact of COVID-19 in adults. *BMJ Paediatr Open* 2020;4(1):e000701. doi: 10.1136/bmjpo-2020-000701 [published Online First: 2020/05/19]
9. Lee J. Mental health effects of school closures during COVID-19. *Rapid Review Lancet* 2020;395:912-20. doi: https://www.thelancet.com/pdfs/journals/lanchi/PIIS2352-4642(20)30109-7.pdf
10. National Youth Trends. Take the Temperature: A national youth trends report understanding the impact of coronavirus on young people in the UK on young people in the UK 2020 [Available from: https://static1.squarespace.com/static/5adf3f90c3c16aec0e1817b9/t/5eb296a85ac37d1ef0096738/1588762425413/Take+the+Temperature+Report+-+National+Youth+Trends.pdf.
11. Education Support. Covid-19 and the classroom: Working in education during the coronavirus pandemic. The impact on education professionals' mental health and wellbeing 2020 [Available from: https://www.educationsupport.org.uk/resources/research-reports/covid-19-and-classroom-working-education-during-coronavirus-pandemic.
12. Panovska-Griffiths J, Kerr CC, Stuart RM, et al. Determining the optimal strategy for reopening schools, the impact of test and trace interventions, and the risk of occurrence of a second COVID-19 epidemic wave in the UK: a modelling study. *The Lancet Child & Adolescent Health*;4(11):817-27. doi: 10.1016/S2352-4642(20)30250-9
13. Viner RM, Bonell C, Drake L, et al. Reopening schools during the COVID-19 pandemic: governments must balance the uncertainty and risks of reopening schools against the clear harms associated with prolonged closure. *Archives of Disease in Childhood* 2020;Published Online First: 03 August 2020. doi: 10.1136/archdischild-2020-319963 doi: 10.1136/archdischild-2020-319963
14. Malterud K, Siersma VD, Guassora AD. Sample Size in Qualitative Interview Studies: Guided by Information Power. *Qual Health Res* 2016;26(13):1753-60. doi: 10.1177/1049732315617444 [published Online First: 2015/11/29]
15. Gale NK, Heath G, Cameron E, et al. Using the framework method for the analysis of qualitative data in multi-disciplinary health research. *BMC Medical Research Methodology* 2013;13(1):117. doi: 10.1186/1471-2288-13-117

Confidential: For Review Only

16. Pearcey S, Shum A, Waite P, et al. COSPACE Study Report 03: Parents/carers report on their own and their children's concerns about children attending school 2020 [Available from: https://emergingminds.org.uk/wp-content/uploads/2020/05/Co-SPACE-report03_School-concerns_23-05-20.pdf accessed 25 June 2020.

17. Todd C, Marchant E, James M, et al. HAPPEN: Return to School Study Initial Report 2020 [Available from: https://happen-wales.co.uk/wp-content/uploads/2020/08/HAPPEN-Return-to-School-Study-Initial-Report-August-2020.pdf.

18. Covid-19 Advisory Sub-group. Report From The Covid-19 Advisory Sub-Group On Education And Children's Issues: Summary Of The Evidence On Children, Schools, Early Learning And Childcare Settings And Transmission From Covid-19 2020 [Available from: https://www.gov.scot/binaries/content/documents/govscot/publications/research-and-analysis/2020/11/coronavirus-covid-19-evidence-on-children-schools-early-learning-and-childcare-settings-and-transmission-from-covid-19---summary-report/documents/advisory-sub-group-on-education-and-childrens-issues---summary-report-of-the-evidence-on-children-schools-early-learning-and-childcare-settings-and-transmission-from-covid-19/advisory-sub-group-on-education-and-childrens-issues---summary-report-of-the-evidence-on-children-schools-early-learning-and-childcare-settings-and-transmission-from-covid-19/govscot%3Adocument/SUMMARY%2BOF%2BTHE%2BEVIDENCE%2BON%2BCHILDREN%2BSCHOOLS%2BEARLY%2BLEARNING%2BAND%2BCHILDCARE%2BSETTINGS%2BAND%2BTRANSMISSION%2BFROM%2BCOVID-19%2B%2528002%2529.pdf.

19. Public Health England. Deaths of people identified as having learning disabilities with COVID-19 in England in the spring of 2020 2020 [Available from: https://assets.publishing.service.gov.uk/government/uploads/system/uploads/attachment_data/file/933612/COVID-19__learning_disabilities_mortality_report.pdf.

20. NHS Digital. Mental Health of Children and Young People in England, 2020: Wave 1 follow up to the 2017 survey 2020 [updated 22/10/2020. Available from: https://digital.nhs.uk/data-and-information/publications/statistical/mental-health-of-children-and-young-people-in-england/2020-wave-1-follow-up accessed 23/10/2020.

21. Kooth. Data release: June 11th 2020. Week 14: How Covid-19 is Affecting the Mental Health of Young People in the BAME Community 2020 [Available from: https://xenzone.com/wp-content/uploads/2020/06/BAME_infographic_June-2020_WEB-v2.pdf

22. Roberts J. Exclusive: 69% of teachers see Covid behaviour slump. *TES* 2020.

23. Murphy J, Spikol E, McBride O, et al. The psychological wellbeing of frontline workers in the United Kingdom during the COVID-19 pandemic: First and second wave findings from the COVID-19 Psychological Research Consortium (C19PRC) Study. *PsyArXiv* 2020:doi:10.31234/osf.io/dcynw.

24. Kim L, E., Leary R, Asbury K. 'We need clear directions, if we're going to move forward. It's as simple as that': Teachers' narratives during partial school reopenings in the COVID-19 pandemic: PsyArZiv; 2020 [Available from: https://psyarxiv.com/m8scj/.

25. Smith LE, Woodland L, Amlôt R, et al. A cross-sectional survey of parental perceptions of COVID-19 related hygiene measures within schools and adherence to social distancing in journeys to and from school. *BMJ Paediatrics Open* 2020;4(1):e000825. doi: 10.1136/bmjpo-2020-000825

26. Harris A, Jones M. COVID 19 – school leadership in disruptive times. *School Leadership & Management* 2020;40(4):243-47. doi: 10.1080/13632434.2020.1811479

27. NAHT. Schools spending tens of thousands of pounds on safety measures without financial support from government 2020 [updated 09/10/20. Available from: https://www.naht.org.uk/news-and-opinion/press-room/schools-spending-tens-of-thousands-of-pounds-on-safety-measures-without-financial-support-from-government/ accessed 12/10/20.

Confidential: For Review Only

28. NIHR ARC East Midlands. Public Perceptions Towards Vaccine Trial Research within Ethnic Minority and Vulnerable Communities 2020 [Available from: http://arc-em.nihr.ac.uk/sites/default/files/field/restricted/Vaccine_Trial_PPI_ARCEM_CRNEM.PDF.
29. Ismail S, A., Saliba V, Bernal JL, et al. SARS-CoV-2 infection and transmission in educational settings: cross-sectional analysis of clusters and outbreaks in England 2020 [Available from: https://assets.publishing.service.gov.uk/government/uploads/system/uploads/attachment_data/file/911267/School_Outbreaks_Analysis.pdf.
30. Wise J. Covid-19: NHS Test and Trace must improve for schools to reopen safely, say researchers. *BMJ* 2020;370:m3083. doi: 10.1136/bmj.m3083
31. Public Health England. Transmission of COVID-19 in school settings and interventions to reduce the transmission: a rapid review [Update 1] 2020 [Available from: https://phe.koha-ptfs.co.uk/cgi-bin/koha/opac-retrieve-file.pl?id=6c419ea93fb3d57f71662fd2fd6be3f6.
32. Faherty LJ, Schwartz HL, Ahmed F, et al. School and preparedness officials' perspectives on social distancing practices to reduce influenza transmission during a pandemic: Considerations to guide future work. *Preventive Medicine Reports* 2019;14:100871. doi: https://doi.org/10.1016/j.pmedr.2019.100871
33. Independent SAGE. Independent SAGE: Learning the lessons from Reopening Schools in Scotland: A sixpoint plan of action 2020 [Available from: https://www.independentsage.org/wp-content/uploads/2020/09/Schools-reopening.pdf accessed 23/10/2020.
34. Bonell C, Melendez-Torres GJ, Viner RM, et al. An evidence-based theory of change for reducing SARS-CoV-2 transmission in reopened schools. *Health & Place* 2020;64:102398. doi: https://doi.org/10.1016/j.healthplace.2020.102398
35. Luttrell A, Petty RE. Evaluations of Self-Focused Versus Other-Focused Arguments for Social Distancing: An Extension of Moral Matching Effects. *Social Psychological and Personality Science*:doi:10.1177/1948550620947853. doi: 10.1177/1948550620947853
36. Drury J, Reicher S, Stott C. COVID-19 in context: Why do people die in emergencies? It's probably not because of collective psychology. *The British journal of social psychology* 2020;59(3):686-93. doi: 10.1111/bjso.12393 [published Online First: 2020/06/17]
37. Coronavirus: Police granted access to details of people told to self-isolate by Test and Trace. *Sky News* 2020 19/10/2020.
38. Bonell C, Michie S, Reicher S, et al. Harnessing behavioural science in public health campaigns to maintain 'social distancing' in response to the COVID-19 pandemic: key principles. *Journal of Epidemiology and Community Health* 2020;74(8):617-19. doi: 10.1136/jech-2020-214290
39. Independent SAGE. Independent SAGE statement on the new UK Government COVID Measures announced on 22nd September 2020 [updated 28/09/2020. Available from: https://www.independentsage.org/independent-sage-statement-on-the-new-uk-government-covid-measures-announced-on-22nd-september/ accessed 23/10/2020.
40. Villa S, Jaramillo E, Mangioni D, et al. Stigma at the time of the COVID-19 pandemic. *Clinical Microbiology and Infection*;https://doi.org/10.1016/j.cmi.2020.08.001 doi: 10.1016/j.cmi.2020.08.001

Confidential: For Review Only

Box 1: Anticipated risk reduction measures at school

The most common measures planned were:

'Bubbles': Groups of students (most commonly year groups) prevented from mixing with other bubbles, and often contained in one part of the school. Separate lunch and break times for bubbles.

Social Distancing: 2m distancing between teachers and students. Distancing not expected between students.

Reduced movement of students around school: students staying in one classroom and teachers moving between classrooms, staggered arrival and leaving, one-way systems, desks facing forward.

Hand sanitiser gel: available throughout school.

Cleaning: Extra cleaning/facilitating cleaning (e.g. removing soft furnishings).

Less common were:

Reduced group sessions: no/reduced assemblies or tutor groups sessions (where these were 'vertical' i.e. included multiple year groups).

Reduced range of subjects: suspending or adapting high Covid-19 risk lessons/activities e.g. music, cooking, science experiments, field trips.

Handwashing reminders: handwashing at certain times of day and posters/reminders to wash hands.

Increased ventilation

Handwashing facilities: Outdoor sinks.

Masks: Rules on wearing face coverings varied as the government guidance changed during data collection, but latterly schools required face coverings to be worn outside of classrooms.

For Review Only

Table 1 Participant characteristics

School	Deprivation quintile (IMD 2019) (1 = least, 5 most)	% of students on free school meals (2020)	% of students with English as an additional language (2018/19)	Families (n)	Staff (n)
S1	2*	under 10%	20-30%	3	5
S2	5*	30-40%	40-50%	1	3
S3	3*	10-20%	30-40%	8	2
S4	3*	20-30%	20-30%	3	0
S5	2*	10-20%	under 10%	0	2
S6	1*	10-20%	10-20%	0	1
S7	2*	under 10%	20-30%	1	0
S8	1~	10-20%	under 10%	0	1
S9	5~	under 10%	under 10%	1	0
S10	3~	10-20%	under 10%	1	0
S11	1~	10-20%	under 10%	0	1
S12	4*	10-20%	10-20%	1	0
S13	1*	10-20%	10-20%	1	0
S14	1*	under 10%	under 10%	1	0

For Review Only

449 **Table 2: Quotes on concern about risk of Covid-19**

Not concerned about Covid-19/risk outweighed by risk of school campuses not reopening	Concerns about Covid-19
"I'm not worried about spreading it...I don't really come in contact with anyone who would be worried" (Emma, yr 8, S1)<br><br>"I'm not worried about illness [Covid-19], per se, I don't think...That's not something that worries me. I'd rather they got... got back to normal, to be honest" (Jess, Mum, S3)<br><br>"if you want the kids back at school, you, you have to accept that... there are risks associated with that" (Georgina, Mum, S3)<br><br>"Resigned acceptance [about school opening] that, that the world has changed and, um, that we kind of just have to get on with things" (Anna, SENCO, S1)	"I'm worried about um – because Jo's Dad is in the kind of very, what's the word I'm looking for? Vulnerable, like medical category 'cause he's only got one functioning lung and my Dad is...has dementia so I'm worried about [Jo] bringing coronavirus home" (Kelly, Mum, S4)<br><br>"I'm a bit concerned. Um, what precaution they're going to take and as Asian background we come on the high risk" (Fatima, Mum, S10)<br><br>"I still would feel awful if I got my partner really ill, you know, um, from my work, when he's allowed to work from home and he's, you know, following all the guidance" (Clare, Teacher, S8)<br><br>"teachers that I've spoken to that have expressed anxieties are teachers who, you know, with things like low-level asthma and, er, other conditions that put them in a position where they may not be COVID fit" (John, Teacher, S3)

450

451

Confidential: For Review Only

Table 3: Quotes about school covid-19 risk reduction measures

**Concerns/barriers**	**Facilitators**
Social distancing impossible	Common good
"I can't see it [social distancing] happening unless there are less children in the school at one time" (Sarah, Mum, S4)<br><br>"you can't properly socially distance in a school so it's a lot of 'that'll do, that'll do' but it's still best it can be" (Matt, Teacher, S3)<br><br>"maybe they [school] have some magical plan, or maybe if they can extend something or something like that, but I... I just feel like there's not enough space for what they're trying to do" (Amber, yr 7, S3)	"I think empowering people to be autonomous and realise that their efforts are, are for the collective good. That they've all got a part to play in it. I think that's much better than, you know, er, publicly shaming them, or or, or criticising them....if you want to achieve compliance and cooperation, it's creating an atmosphere that it's in everyone's best interest and you contribute to the common good" (John, Teacher, S3)
Concerns about year bubbles	Messaging and reminders
"at the end of the day, we're all gonna walk out of school at the same time and we're all just gonna walk together and end up in the same place " (Amber, yr 7, S3)<br><br>"siblings they are mixing bubbles, the bubbles become 500, 700 [people], um, so it's a gesture" (Claire, Teacher, S8)	"if there's a consistent message, 'this is just what you do now, if you can', then, um, then they'll just pick it up really and get on with it" (Georgina, Mum, S3)<br><br>"I think having more visuals is really quite key so there's, they are reminded of what's, what's going on. Um, I don't feel like we have enough at the moment, to be honest" (Julia, Teacher, S11)
Concerns about hand-hygiene	Staff culture shift
"I suspect we're going to run out of hand sanitisers in the, um, the ones that are kind of communally put up around the school …I'd say that they, they're going to run out by break time" (Julia, Teacher, S11)<br><br>"They [school bathrooms] never have soap or anything, like, loads of the taps don't work!" (Daisy, yr 7, S3)	"our biggest weapon will be 'don't be in work if you're ill'" (Jackie, Head, S1)
Lack of compliance with new rules	
"[on handwashing] maybe forgetting or teachers not reminding the students enough or them being too lazy or maybe their friends might be going somewhere but their	

Confidential: For Review Only

friends don't wanna wait for them so they just can't be bothered to wash their hands" (Ali, yr 10, S13)<br><br>"I feel like, you know, people are just are not gonna do it [social distance]. They're just gonna do what they want and like run around the whole place " (Amber, yr 7, S3)<br><br>"a line that's been put on the floor that shows the two metres to the teacher. Um, what's interesting there, of course some students are going to see that as a bit of an invite or a challenge to cross that line, literally" (Tracey, Mum, S3)<br><br>"[my child] might just go to the sink, wash the few fingers at the front and then leave. We don't actually know if they're going to be doing the full happy birthday… it baffles me how it's [enforcing handwashing] actually going to work" (Samira, Mum, S4)	
Specific groups	
"disadvantaged students, and students that would normally get more support in a lesson - because it's more like lecture style now, so we're not really allowed to walk between the desks or anything - those students are going to miss out, the ones who wouldn't normally put their hand up " (Finn, Teacher, S5)<br><br>"the young people … with the highest level of needs, who've got education health and care plans …might not fully understand the need for personal space, or…we've just added this whole extra kind of layer of complexity into their social world that I'm sure is going to be hugely challenging" (Anna SENCO, S1)	

Confidential: For Review Only

Table 4: Quotes about school management of Covid-19 cases

**Reactions to Covid-19 cases**
"if someone coughed in my class, I would see one or two people shying away from them a bit and that most of the class is laughing a little bit and saying, ‘oh you have Corona virus’ as a joke" (David, yr 8, S3)<br><br>"I think everyone would just be very pragmatic about it and I can’t see there being panic stations" (John, Teacher, S3)
**Reporting symptoms**
"I think it will be very tricky… for parents to differentiate… fever and cough, they are quite common symptoms kids get in the winter season " (Sangita, Mum, S13)
**Test-and-trace**
"If there was a risk we were going to get the virus and it would make everyone safer then I would do it [regular testing]" (Jasmine, yr 10, S4)<br><br>“I understand the need [to collect data] but on the same token, it’s just that personal data being collected about my child makes me feel very uncomfortable” (Sarah, mum, S4)<br><br>“market it towards the fact that they’ve missed so much education, this is something that can potentially help keep them in school for as long as possible. Because that’s ultimately what I think most parents are concerned about” (Dan, Head of yr 8, S2)

Confidential: For Review Only

BMJ Paediatrics Open

Reducing Covid-19 risk in schools: a qualitative examination of secondary school staff and familyi views and concerns in the South West of England

Journal:	*BMJ Paediatrics Open*
Manuscript ID	bmjpo-2020-000987.R1
Article Type:	Original research
Date Submitted by the Author:	09-Jan-2021
Complete List of Authors:	Lorenc, Ava; University of Bristol,<br>Kesten, Joanna; University of Bristol School of Social and Community Medicine, NIHR Collaboration for Leadership in Applied Health Research and Care West and NIHR Health Protection Research Unit in Evaluation of Interventions<br>Kidger, Judi; University of Bristol<br>Langford, Rebecca; University of Bristol<br>Horwood, Jeremy; University of Bristol
Keywords:	COVID-19

SCHOLARONE™
Manuscripts

I, the Submitting Author has the right to grant and does grant on behalf of all authors of the Work (as defined in the below author licence), an exclusive licence and/or a non-exclusive licence for contributions from authors who are: i) UK Crown employees; ii) where BMJ has agreed a CC-BY licence shall apply, and/or iii) in accordance with the terms applicable for US Federal Government officers or employees acting as part of their official duties; on a worldwide, perpetual, irrevocable, royalty-free basis to BMJ Publishing Group Ltd ("BMJ") its licensees and where the relevant Journal is co-owned by BMJ to the co-owners of the Journal, to publish the Work in this journal and any other BMJ products and to exploit all rights, as set out in our licence.

The Submitting Author accepts and understands that any supply made under these terms is made by BMJ to the Submitting Author unless you are acting as an employee on behalf of your employer or a postgraduate student of an affiliated institution which is paying any applicable article publishing charge ("APC") for Open Access articles. Where the Submitting Author wishes to make the Work available on an Open Access basis (and intends to pay the relevant APC), the terms of reuse of such Open Access shall be governed by a Creative Commons licence – details of these licences and which Creative Commons licence will apply to this Work are set out in our licence referred to above.

Other than as permitted in any relevant BMJ Author's Self Archiving Policies, I confirm this Work has not been accepted for publication elsewhere, is not being considered for publication elsewhere and does not duplicate material already published. I confirm all authors consent to publication of this Work and authorise the granting of this licence.

Confidential: For Review Only

Reducing Covid-19 risk in schools: a qualitative examination of secondary school staff and family views and concerns in the South West of England

Dr Ava Lorenc, Senior Research Associate[1, 2]

Dr Joanna M Kesten, Research Fellow [1, 2, 3]

Dr Judi Kidger, Lecturer[2]

Dr Rebecca Langford, Research Fellow[2]

Dr Jeremy Horwood, Associate Professor [1, 2, 3] (corresponding author; j.horwood@bristol.ac.uk)

1. National Institute for Health Research Applied Research Collaboration West (NIHR ARC West), University Hospitals Bristol and Weston NHS Foundation Trust, 9th Floor, Whitefriars, Lewins Mead, Bristol, BS1 2NT, UK.
2. Population Health Sciences, Bristol Medical School, University of Bristol, Canynge Hall, 39 Whatley Road, Bristol, BS8 2PS, UK
3. NIHR Health Protection Research Unit (HPRU) in Behavioural Science and Evaluation at University of Bristol Canynge Hall, 39 Whatley Road, Bristol, BS8 2PS, UK

Word count: 3070 (please see cover letter regarding this)

Abstract

Objective To investigate student, parent/carer, and secondary school staff attitudes towards school Covid-19 mitigation measures.

Methods Recruitment used school communication, community organisations and snowball sampling in South West England. Audio recorded online or phone individual/group semi-structured interviews were conducted in July-Sept 2020 and lasted 30-60 minutes. Interviews focused on views towards social distancing, hand-hygiene and testing. Framework analysis was performed on interview notes/transcripts.

Results

Participants were 15 staff, 20 parents and 17 students (11-16 years) from 14 diverse schools. Concerns about Covid-19 risk at school, especially to vulnerable individuals, were outweighed by perceived risks of missed learning. Some staff felt guilt around being a potential “spreader” by teaching multiple classes. Findings highlighted a wide variety of school covid-19 mitigation measures being deployed due to ambiguous government guidance. Participants generally saw mitigation

Confidential: For Review Only

measures as an acceptable and pragmatic solution to the perceived impossibility of social distancing in crowded schools, although anticipated challenges changing habitual behaviour. Participants supported school Covid-19 testing but identified the need to consider data security and stigma around Covid-19 diagnosis. Staff were concerned about unintended consequences of risk-reduction strategies on student behaviour, learning, and pastoral care, particularly for those with Special Educational Needs or mental health issues who may find the measures especially challenging, and resultant widening inequalities.

Conclusion

Families and staff supported Covid-19 mitigation measures in schools and would welcome the roll out school Covid-19 testing. Clear messaging and engendering collective responsibility are important for compliance and success of Covid-19 mitigation measures. However, schools and policymakers should consider unintended consequences of measures, providing extra support for vulnerable students and those with additional needs, and consider ways to avoid widening educational and health inequalities. Findings demonstrate the acceptability of school Covid-19 infection control measures is likely to be influenced by the balance of risks and benefits to students.

Confidential: For Review Only

What is known about the subject

- Schools reopened in September 2020 with Covid-19 risk reduction measures in place. Compliance with and impact of these measures – especially on vulnerable groups - is unclear.
- Current UK government Covid-19 policy includes punitive measures to ensure behavioural compliance.
- Scientists have advised scaling-up testing and tracing within schools

What this study adds

- Given the challenges of social distancing under current government school guidelines, families and staff view effective testing and innovative risk reduction measures as imperative
- Schools should adopt an encouraging and compassionate approach to implementing risk reduction measures, with clear messaging emphasising collective responsibility.
- Families and staff support schools remaining open but concerns over unintended consequences of risk reduction measures must be addressed by schools and policy makers.

Introduction

To reduce the spread of Covid-19, a UK 'lockdown' was announced on 23rd March 2020 and school campuses, as in most countries worldwide[1], were closed to all but vulnerable or priority students. Prior to the summer holidays some year groups, including year 10, returned to school for face-to-face teaching. In July, the government announced all school campuses would fully reopen in September.

Evidence for school closures reducing infection spread is equivocal [2]. Those in favour of school campuses reopening to all pointed to the impact on learning [3], particularly for lower socioeconomic groups [4], widening inequalities and consequences for students' physical and mental health [1 5-10]. Remote learning also affected staff wellbeing and mental health [11]. However, schools reopening carried risks - modelling suggested increased Covid-19 infection would occur if schools reopened full time (combined with easing of other restrictions and without scaled up school testing) [12].

Government guidance set out a range of school-based protective measures to reduce Covid-19 outbreaks, including hand-hygiene, cleaning, reporting symptoms, and social distancing. Further, a UK modelling study recommended existing testing should be scaled up [12 13].

Understanding the views and concerns of school staff, parents and students about the implementation of this guidance is key to ensuring compliance, avoiding unintended harms[13] and supporting schools to reduce risks. This is especially important in secondary schools as the risk of transmission in teenagers is higher than young children, Covid-19 measures particularly impact exam years, and secondary school students are more concerned than primary about Covid-19[14]. This

https://mc.manuscriptcentral.com/bmjpo

qualitative study undertaken in secondary schools aimed to rapidly explore student, parent/carer and school staff attitudes towards school Covid-19 mitigation measures, views on managing Covid-19 infections in schools and opinions about student groups who may be particularly affected by these measures.

Methods

Study setting

The study was conducted during the school summer holidays immediately following school campus closures and the first week of the new academic year (between July and September 2020), with 11-16 year olds, parents and school staff recruited via secondary schools and local community organisations in the South West of England.

Sampling and recruitment

Because lower socioeconomic and Black, Asian and Minority Ethnic (BAME) populations are disproportionately affected by Covid-19, we contacted 21 secondary schools with relatively higher levels of these populations (based on school-level data from local Public Health departments) to ensure we captured their specific concerns. Participating schools sent study information to potential participants (e.g. by email/newsletter). We included staff (teachers, senior leaders/headteachers, Special Educational Needs Coordinators (SENCOs), or those with a role in infection control), students and their parents/carers, at Bristol secondary state schools. We focused student/parent recruitment on Year 8 (age 12-13; most had no face-to-face teaching since March) and Year 10 (age 14-15; had briefly returned to school campus prior to the summer holidays; faced key exams next year). We also advertised the study via community organisations (e.g. BAME support groups, youth groups) in areas of higher BAME groups/deprivation (newsletters, social media, and direct contact with members) and used snowball sampling (including families inviting friends to be interviewed with them or separately). Individuals interested in participating contacted the researcher directly to arrange an interview. All eligible volunteers were interviewed (none refused or dropped out).

Interviews

Participants chose to be interviewed on their own, with a friend and/or parent (students) or with colleagues (staff). AL (an experienced qualitative researcher) led interviews by phone or video, with JMK or JK present for group interviews/student interviews without a parent present. Interviews were 30-60 minutes and audio recorded. Participants provided audio-recorded verbal informed consent (or assent and parent consent for those under 16 years).

Confidential: For Review Only

97 Students and school staff provided input into the study design and topic guides.

98 Topic guides (see Supplementary materials) were tailored for staff or families, but both covered attitudes towards UK recommendations at the time (social distancing, school hand-hygiene and infection control strategies), and acceptability of school Covid-19 testing, and were used flexibly, allowing exploration of issues raised by participants. We offered participants a shopping voucher as thanks for their time (£10 for each family member; £20 for staff).

103 Data collection was informed by the concept of information power[15] and pragmatic considerations of the project timeline.

105 **Patient and public involvement**

106 The NIHR ARC West Young Peoples Advisory Group provided feedback on study design and interview topic guides.

108 **Analysis**

109 Producing timely reports for local and national stakeholders necessitated rapid analysis (reports available at https://arc-w.nihr.ac.uk/research/projects/the-back-to-school-study). The framework method[16] was used to analyse the data. AL used anonymised interview notes to develop an initial coding framework in Microsoft EXCEL. Codes reflecting the topic guide headings and inductive coding were combined to produce overarching themes. Data within each code were summarised. The framework was further developed using verbatim anonymised interview transcripts. Each author independently read through a subset of interviews and added new codes and quotes to the existing framework, discussing these additions as a team. Framework analysis was appropriate for the specific a priori questions and limited time frame.

118 The study was approved by the Faculty of Health Sciences Committee for Research Ethics, University of Bristol (ref 108084).

Confidential: For Review Only

Results

Participants

Three of the 21 contacted schools participated by sending information to potential participants, with participants from an additional 11 schools recruited via other methods. We did not record how participants heard about the study. Participants were 15 school staff (Heads/Assistant Heads, teachers, SENCOs) from seven schools, and 20 families from ten schools (12 with a BAME child/parent) - 17 students (9 females; 8 males; 6 yr 7s, 4 yr8s, 2 yr9s, 5 yr 10s) and 20 parents (19 mothers, 1 father) (Table 1). For family interviews 9 were one parent and one child (one where child was interviewed alone so an additional researcher was present), 6 one parent only, 3 one parent and two children, and one was two families together (friends and one parent each). All five year 10 students had attended school for a few days, and all staff participants had been working during lockdown (some of whom had worked within the school environment).

Covid-19 risk concerns

Most staff and around half of students and parents (hereafter referred to as families where there is agreement in views) were not concerned about personal/family Covid-19 risk from returning to school (Table 2). However, many staff anticipated increased cases describing schools as "petri dishes". Some staff worried about being a potential "spreader" (Table 2). Staff also had concerns about higher risk staff and students, including those from BAME backgrounds, those who were pregnant or who had health conditions.

Many families were more concerned about the negative educational consequences of students not being at school, accepting that home schooling cannot continue indefinitely. However, families did note the risk to vulnerable family members (due to health or age) (Table 2). Concern was more common in families with BAME members, although a minority explicitly cited ethnicity-related risk, with some noting the lack of scientific understanding around this risk.

School risk reduction measures

Outside school, most students had accepted social distancing, wearing masks and handwashing, understanding their necessity, with minor negative comments such as social distancing being "not nice". Most aimed to socially distance and some had almost no social contact outside their household during the first lockdown, some enjoying staying home.

https://mc.manuscriptcentral.com/bmjpo

Confidential: For Review Only

Interviewees highlighted a wide variety of school Covid-19 risk reduction measures (see Box 1). Participants agreed such measures were needed, although staff were frustrated with the lack of detail in government guidance, meaning each school had to develop their own plans. Staff were generally happy with schools' plans but had reservations about feasibility and how they would "play out in practice".

Barriers/concerns regarding risk reduction measures

All participants agreed student social distancing was impossible and "pointless" given the numbers of people and lack of space (Table 3). Year 'bubbles' (see Box 1) were seen as a "pragmatic solution", although there was concern about crossover via siblings or groups leaving school together, and teachers (though less commonly reported). In terms of compliance with risk reduction measures, of most concern among families was forgetting, and a desire to "be normal". A few staff were concerned about rule enforcement methods.

Staff and family concerns about hand-hygiene/infection control were mostly practical, including: availability of resources (sanitiser/soap, sinks, cleaners) - one school estimated a £40k cost of hand sanitiser; bathroom cleanliness; time for handwashing; and effective use of measures e.g. hand sanitiser vs. washing, or proper use of masks. Some were concerned about ventilation e.g. windows not opening.

A minority of staff were worried about behavioural issues arising from students having to stay in the same classroom e.g. unsupervised lesson changeovers, student boredom and lack of movement. Other concerns included risk of using public transport and reduced range of lessons/activities. An important concern for staff was the impact of these measures on learning and pastoral care, especially social distancing measures e.g. fewer interactive lessons, and less opportunity to support individual students.

Staff were concerned social distancing measures would particularly affect students with special educational needs (SEN) or mental health issues. Particular issues for students with SEN included (Table 3): struggling to understand and comply with the changes; finding less interactive lessons challenging; SEN staff being unable to work physically closely with students (and lack of PPE for this); physical needs e.g. personal care or feeding; and removal of 'safe spaces' (due to bubbles and infection risk from soft furnishing).

https://mc.manuscriptcentral.com/bmjpo

Facilitators/suggestions regarding risk reduction measures

The main suggestion from staff for facilitating the new rules was educating students about their importance, and encouraging a “we culture” of collective responsibility through a supportive, considerate approach (Table 3), although a minority of families thought handwashing should be compulsory and enforced.

Staff and parents suggested clear consistent messaging and daily reminders, both verbal and visual. Clear and regular communication from schools about the measures would also reassure parents (Table 3).

Other staff suggestions to support the mitigation measures included:

- Funding for cleaning products, PPE and hand-sanitiser; additional classroom equipment to ensure no sharing
- Government guidance on PPE and students with SEN
- Staff training on how to work within the new measures
- Risk assessments for vulnerable students/staff
- Shorter lessons to allow time for handwashing
- Students bringing their own hand-sanitiser
- Encouraging staff to stay home if displaying symptoms (a culture shift from presenteeism common in schools) (Table 3).

School management of Covid-19 cases

Reaction to Covid-19 cases

Several families, mostly BAME families, anticipated possible stigma around Covid-19 diagnosis. Staff did not generally anticipate stigma, due to increasing understanding of Covid-19 among students, and school communities having a generally tolerant and accepting attitude due to existing school population diversity - although two mentioned possible "mass hysteria" and another that teenagers "love to joke and point a finger" (see Table 4).

Reporting symptoms to school

Most families and some staff anticipated under-reporting to school of student Covid-19 symptoms, due to embarrassment, wanting to attend school, or parents needing to work. For staff symptoms, underreporting reasons included a culture of presenteeism, and guilt at having exposed themselves to risk. Families wanted clarity about symptoms to report, method of reporting and implications (see Table 4).

Confidential: For Review Only

School Covid-19 testing

All staff and most parents thought testing in schools was important and would be enthusiastic about monthly testing. Testing at schools would reassure students, parents, and staff about school safety, and encourage attendance - a "massive selling point for schools".

A minority of participants had concerns about school testing including:

- Parental concerns about use and anonymity of information, particularly among BAME families (see Table 4).
- More school closures due to cases detected by testing, with implications for parents (time off work), students (loss of learning) and schools (attendance figures and academic achievement).
- Feasibility of testing the whole school – time, space and administration needed.

Staff suggested emphasising to families the importance of testing, including a potentially reduced risk of whole school closure (see Table 4). They also emphasised the need for quick and minimally disruptive testing. Families wanted care, discretion and anonymity in notification of positive results.

Confidential: For Review Only

Discussion

Families and school staff had concerns about an increased Covid-19 risk with the full reopening of schools, particularly to vulnerable individuals, but on balance most felt the benefits outweighed the risks. Some staff anticipated guilt at their potential to spread Covid-19. Students, their parents and school staff generally felt planned school risk reduction measures were acceptable and pragmatic solutions to the perceived impossibility of social distancing between students in crowded schools. Negative unintended consequences of the new measures were anticipated on student behaviour, learning, pastoral care, and particularly for those with SEN or mental health issues who may find the measures especially challenging. Stigma related to Covid-19 positivity was thought unlikely to be widespread in schools by staff, although families, particularly BAME families, anticipated possible stigma around Covid-19 diagnosis. Case reporting must therefore be managed sensitively. The imperative for testing in schools was recognised by staff and most parents, albeit with concerns over data security and feasibility.

Contrasting with previous data [14], students, parents and staff understood the need for risk reduction measures. Staff concerns about negative unintended consequences of the pandemic/risk reduction measures are shared by other UK teachers, policymakers, and parents[6 14 17 18]. Concerns have been raised particularly in relation to deprived communities and SEN students, with some evidence of a disproportionate effect on people with learning disabilities (in terms of Covid-19 deaths)[19], worsening mental health among children[20] especially those from BAME backgrounds[21], and worsening student behaviour[22]. The impact on teachers appears similar to other frontline workers, with high anxiety, depression and distress levels, partly related to feelings of guilt at potentially infecting others and conflicting duties[23 24]. In addition to previously noted feasibility concerns over risk reduction measures [25 26], we identified issues of year-group ‘bubble’ crossover and non-compliance due to forgetting and wanting to be ‘normal’/socialise and social distancing being impossible due to crowded school sites. The barriers we identified support calls for funding to help implement risk reduction measures (including hand-sanitiser, extra cleaning, free masks for all pupils and hiring extra teachers and teaching rooms so smaller class sizes can enable social distancing) [17 27], with schools reportedly spending up to £8k each on Covid-19 risk reduction measures in early reopening of campuses [28]. BAME parents’ concerns about use of data for school Covid-19 testing echo scepticism and apprehension among BAME public contributors around Covid-19 vaccine trials [29].

Limitations

Confidential: For Review Only

This study was conducted in a limited time frame in response to an urgent need to understand attitudes towards school Covid-19 mitigation measures and views on managing infections in schools. As a result, we took a pragmatic approach, capturing one timepoint in a rapidly changing field. Several limitations must be acknowledged. During the study, the community Covid-19 transmission rates in the research setting were relatively low; this context may have influenced participants risk perceptions. Furthermore, the sample lacks those with English as an additional language (recruitment materials were translated); those disengaged from school (recruitment was mainly via schools); and those without internet access/computers (due to Covid-19 restrictions we mainly used online recruitment). In addition, the majority of parents interviewed were mothers. Our findings have identified useful insights with important implications for schools, but should be interpreted with these limitations in mind.

Implications

Due to lack of detailed school guidance, our interviews highlighted a wide variety of school Covid-19 mitigation measures, meaning comparison of effectiveness of mitigation measures will be difficult.

More could be done to reduce school transmission, such as reducing crowding to improve social distancing (e.g. using other community spaces) and introducing effective school Covid-19 testing[30-33]. These actions are even more vital as the pandemic progresses and family and staff opinions about the benefits of schools remaining open may shift. Even with the implementation of such measures, as demonstrated in the UK in early 2021, stopping face-to-face teaching may be a necessary last resort to reduce covid-19 transmission.

Barriers to mitigation measure compliance may be addressed through clear, consistent information and reminders[24 33], and engendering a sense of collective responsibility [34]. This applies both to communication from schools to families and from government to schools - our findings demonstrate that government have provided ambiguous recommendations rather than clear instructions which has left schools having to interpret them. Clear communication is needed from government based on latest scientific evidence. Our findings also demonstrate the importance of emphasising to students the collective good, benefits for their family or wider community, and a sense of collective identity and responsibility[35-37]. This conflicts with UK government use of police and fines to enforce measures in wider society[38], for which behavioural scientists have expressed concern[39 40]. Schools may need policies, rules and reward systems, and discipline [22 35] but should consider a compassionate approach to Covid-19 mitigation measures, which seeks to promote collective identity and social norms, as well as being sensitive to potential stigma of Covid-19[41].

Confidential: For Review Only

Although our findings support schools returning to face-to-face teaching for all (risks from school closure outweigh those of Covid-19 [18]), policymakers must consider potential unintended consequences of measures, and ways to help schools support vulnerable individuals and those with additional needs and avoid widening inequalities [24]. This may include additional funding and resources to ensure that staff and pupils can adhere to the current government advice [42], and access to home learning resources for the most vulnerable. It may also involve training staff to meet the emotional and mental health needs of students [6]; for example many organisations are suggesting a trauma informed approach for schools during this period.

Future research

Further research is needed to understand the welfare and morale of staff and students as the pandemic continues to disrupt schools, including the mental and physical impact of self-isolation of groups of students, and the impact on learning and disruption to schools. Further research to identify the ongoing disproportionate impact of the Covid-19 pandemic, both within and outside schools, on vulnerable and deprived groups is vital. There is also a need for research to understand how to address BAME families' particular concerns relating to Covid-19 transmission within schools.

Conclusion

Families and staff support Covid-19 mitigation measures in schools as a means of students having face-to-face education. Clear messaging and engendering collective responsibility are important for compliance and success of mitigation measures. However, schools and policymakers need to consider potential unintended consequences of measures, ways to support vulnerable individuals and those with additional needs, and avoidance of widening inequalities. Additional funding may be required.

Funding

This work was supported by National Institute for Health Research, Applied Research Collaboration West (NIHR ARC West) and NIHR Health Protection Research Unit in Behavioural Science and Evaluation (NIHR HPRU BSE). AL, JMK and JH's time was supported by NIHR ARC West and NIHR HPRU BSE. JK's time was supported by NIHR School for Public Health Research. The views expressed are those of the authors and not necessarily those of the NIHR, the Department of Health and Social Care.

Confidential: For Review Only

Competing interests

No authors have any conflict of interest to declare.

Author contributions

All authors have been involved in drafting the paper, read and approved the final version. AL recruited participants, collected and analysed data and led the writing of this manuscript. JK and JMK assisted with data collection. All authors collaborated on analysis.

Data sharing

Interview transcripts are available on request from University of Bristol Research Data Storage Facility.

Acknowledgements

We would like to thank the valuable contribution of our participants and the community groups who helped with recruitment: Baggator; Barton Hill Activity Club; Black South West Network; Bristol Somali Voice; Bristol Somali Forum; BS5 Secondary Education Forum; The Care Forum; Felix Road Adventure Playground; Off The Record; St George Community Centre; Ujima Radio; Up Our Street; Young Bristol. We are also grateful to the ARC West YPAG and teachers who were involved in designing the study.

Confidential: For Review Only

References

1. Crawley E, Loades M, Feder G, et al. Wider collateral damage to children in the UK because of the social distancing measures designed to reduce the impact of COVID-19 in adults. *BMJ Paediatr Open* 2020;4(1):e000701. doi: 10.1136/bmjpo-2020-000701 [published Online First: 2020/05/19]

2. Viner RM, Russell SJ, Croker H, et al. School closure and management practices during coronavirus outbreaks including COVID-19: a rapid systematic review. *The Lancet Child & Adolescent Health* 2020;4(5):397-404. doi: https://doi.org/10.1016/S2352-4642(20)30095-X

3. Garbe A, Ogurlu U, Logan N, et al. COVID-19 and Remote Learning: Experiences of Parents with Children during the Pandemic. *American Journal of Qualitative Research* 2020;4(3):45-65.

4. Pensiero N, Kelly A, Bokhove C. Learning inequalities during the Covid-19 pandemic: how families cope with home-schooling 2020 [Available from: https://www.southampton.ac.uk/publicpolicy/covid19/learning-inequalities-covid-19.page.

5. Armitage R, Nellums LB. Considering inequalities in the school closure response to COVID-19. *The Lancet Global Health* 2020;8(5):e644. doi: 10.1016/S2214-109X(20)30116-9

6. Lundie D, Law J. Teachers' Responses and Expectations in the COVID-19 School Shutdown Period in the UK: University of Glasgow; 2020 [Available from: http://eprints.gla.ac.uk/221329/.

7. Patrick SW, Henkhaus LE, Zickafoose JS, et al. Well-being of Parents and Children During the COVID-19 Pandemic: A National Survey. *Pediatrics* 2020:e2020016824. doi: 10.1542/peds.2020-016824

8. Van Lancker W, Parolin Z. COVID-19, school closures, and child poverty: a social crisis in the making. *The Lancet Public Health* 2020;5(5):e243-e44. doi: 10.1016/S2468-2667(20)30084-0

9. Lee J. Mental health effects of school closures during COVID-19. *Rapid Review Lancet* 2020;395:912-20. doi: https://www.thelancet.com/pdfs/journals/lanchi/PIIS2352-4642(20)30109-7.pdf

10. National Youth Trends. Take the Temperature: A national youth trends report understanding the impact of coronavirus on young people in the UK on young people in the UK 2020 [Available from: https://static1.squarespace.com/static/5adf3f90c3c16aec0e1817b9/t/5eb296a85ac37d1ef0096738/1588762425413/Take+the+Temperature+Report+-+National+Youth+Trends.pdf.

11. Education Support. Covid-19 and the classroom: Working in education during the coronavirus pandemic. The impact on education professionals' mental health and wellbeing 2020 [Available from: https://www.educationsupport.org.uk/resources/research-reports/covid-19-and-classroom-working-education-during-coronavirus-pandemic.

12. Panovska-Griffiths J, Kerr CC, Stuart RM, et al. Determining the optimal strategy for reopening schools, the impact of test and trace interventions, and the risk of occurrence of a second COVID-19 epidemic wave in the UK: a modelling study. *The Lancet Child & Adolescent Health*;4(11):817-27. doi: 10.1016/S2352-4642(20)30250-9

13. Viner RM, Bonell C, Drake L, et al. Reopening schools during the COVID-19 pandemic: governments must balance the uncertainty and risks of reopening schools against the clear harms associated with prolonged closure. *Archives of Disease in Childhood* 2020;Published Online First: 03 August 2020. doi: 10.1136/archdischild-2020-319963 doi: 10.1136/archdischild-2020-319963

14. Pearcey S, Shum A, Waite P, et al. COSPACE Study Report 03: Parents/carers report on their own and their children's concerns about children attending school 2020 [Available from: https://emergingminds.org.uk/wp-content/uploads/2020/05/Co-SPACE-report03_School-concerns_23-05-20.pdf accessed 25 June 2020.

15. Malterud K, Siersma VD, Guassora AD. Sample Size in Qualitative Interview Studies: Guided by Information Power. *Qual Health Res* 2016;26(13):1753-60. doi: 10.1177/1049732315617444 [published Online First: 2015/11/29]

Confidential: For Review Only

16. Gale NK, Heath G, Cameron E, et al. Using the framework method for the analysis of qualitative data in multi-disciplinary health research. *BMC Medical Research Methodology* 2013;13(1):117. doi: 10.1186/1471-2288-13-117

17. Todd C, Marchant E, James M, et al. HAPPEN: Return to School Study Initial Report 2020 [Available from: https://happen-wales.co.uk/wp-content/uploads/2020/08/HAPPEN-Return-to-School-Study-Initial-Report-August-2020.pdf.

18. Covid-19 Advisory Sub-group. Report From The Covid-19 Advisory Sub-Group On Education And Children's Issues: Summary Of The Evidence On Children, Schools, Early Learning And Childcare Settings And Transmission From Covid-19 2020 [Available from: https://www.gov.scot/binaries/content/documents/govscot/publications/research-and-analysis/2020/11/coronavirus-covid-19-evidence-on-children-schools-early-learning-and-childcare-settings-and-transmission-from-covid-19---summary-report/documents/advisory-sub-group-on-education-and-childrens-issues---summary-report-of-the-evidence-on-children-schools-early-learning-and-childcare-settings-and-transmission-from-covid-19/advisory-sub-group-on-education-and-childrens-issues---summary-report-of-the-evidence-on-children-schools-early-learning-and-childcare-settings-and-transmission-from-covid-19/govscot%3Adocument/SUMMARY%2BOF%2BTHE%2BEVIDENCE%2BON%2BCHILDREN%2BSCHOOLS%2BEARLY%2BLEARNING%2BAND%2BCHILDCARE%2BSETTINGS%2BAND%2BTRANSMISSION%2BFROM%2BCOVID-19%2B%2528002%2529.pdf.

19. Public Health England. Deaths of people identified as having learning disabilities with COVID-19 in England in the spring of 2020 2020 [Available from: https://assets.publishing.service.gov.uk/government/uploads/system/uploads/attachment_data/file/933612/COVID-19__learning_disabilities_mortality_report.pdf.

20. NHS Digital. Mental Health of Children and Young People in England, 2020: Wave 1 follow up to the 2017 survey 2020 [updated 22/10/2020. Available from: https://digital.nhs.uk/data-and-information/publications/statistical/mental-health-of-children-and-young-people-in-england/2020-wave-1-follow-up accessed 23/10/2020.

21. Kooth. Data release: June 11th 2020. Week 14: How Covid-19 is Affecting the Mental Health of Young People in the BAME Community 2020 [Available from: https://xenzone.com/wp-content/uploads/2020/06/BAME_infographic_June-2020_WEB-v2.pdf

22. Roberts J. Exclusive: 69% of teachers see Covid behaviour slump. *TES* 2020.

23. Murphy J, Spikol E, McBride O, et al. The psychological wellbeing of frontline workers in the United Kingdom during the COVID-19 pandemic: First and second wave findings from the COVID-19 Psychological Research Consortium (C19PRC) Study. *PsyArXiv* 2020:doi:10.31234/osf.io/dcynw.

24. UNESCO. School reopening: ensuring learning continuity 2020 [Available from: https://unesdoc.unesco.org/ark:/48223/pf0000373610 accessed 6th January 2021.

25. Kim L, E., Leary R, Asbury K. 'We need clear directions, if we're going to move forward. It's as simple as that': Teachers' narratives during partial school reopenings in the COVID-19 pandemic: PsyArZiv; 2020 [Available from: https://psyarxiv.com/m8scj/.

26. Smith LE, Woodland L, Amlôt R, et al. A cross-sectional survey of parental perceptions of COVID-19 related hygiene measures within schools and adherence to social distancing in journeys to and from school. *BMJ Paediatrics Open* 2020;4(1):e000825. doi: 10.1136/bmjpo-2020-000825

27. Harris A, Jones M. COVID 19 – school leadership in disruptive times. *School Leadership & Management* 2020;40(4):243-47. doi: 10.1080/13632434.2020.1811479

28. NAHT. Schools spending tens of thousands of pounds on safety measures without financial support from government 2020 [updated 09/10/20. Available from: https://www.naht.org.uk/news-and-opinion/press-room/schools-spending-tens-of-

Confidential: For Review Only

thousands-of-pounds-on-safety-measures-without-financial-support-from-government/ accessed 12/10/20.

29. NIHR ARC East Midlands. Public Perceptions Towards Vaccine Trial Research within Ethnic Minority and Vulnerable Communities 2020 [Available from: http://arc-em.nihr.ac.uk/sites/default/files/field/restricted/Vaccine_Trial_PPI_ARCEM_CRNEM.PDF.

30. Ismail S, A., Saliba V, Bernal JL, et al. SARS-CoV-2 infection and transmission in educational settings: cross-sectional analysis of clusters and outbreaks in England 2020 [Available from: https://assets.publishing.service.gov.uk/government/uploads/system/uploads/attachment_data/file/911267/School_Outbreaks_Analysis.pdf.

31. Wise J. Covid-19: NHS Test and Trace must improve for schools to reopen safely, say researchers. *BMJ* 2020;370:m3083. doi: 10.1136/bmj.m3083

32. Public Health England. Transmission of COVID-19 in school settings and interventions to reduce the transmission: a rapid review [Update 1] 2020 [Available from: https://phe.koha-ptfs.co.uk/cgi-bin/koha/opac-retrieve-file.pl?id=6c419ea93fb3d57f71662fd2fd6be3f6.

33. World Health Organization. Considerations for school-related public health measures in the context of COVID-19 2020 [Available from: https://www.who.int/publications/i/item/considerations-for-school-related-public-health-measures-in-the-context-of-covid-19 accessed 6th January 2021.

34. Faherty LJ, Schwartz HL, Ahmed F, et al. School and preparedness officials' perspectives on social distancing practices to reduce influenza transmission during a pandemic: Considerations to guide future work. *Preventive Medicine Reports* 2019;14:100871. doi: https://doi.org/10.1016/j.pmedr.2019.100871

35. Bonell C, Melendez-Torres GJ, Viner RM, et al. An evidence-based theory of change for reducing SARS-CoV-2 transmission in reopened schools. *Health & Place* 2020;64:102398. doi: https://doi.org/10.1016/j.healthplace.2020.102398

36. Luttrell A, Petty RE. Evaluations of Self-Focused Versus Other-Focused Arguments for Social Distancing: An Extension of Moral Matching Effects. *Social Psychological and Personality Science*:doi:10.1177/1948550620947853. doi: 10.1177/1948550620947853

37. Drury J, Reicher S, Stott C. COVID-19 in context: Why do people die in emergencies? It's probably not because of collective psychology. *The British journal of social psychology* 2020;59(3):686-93. doi: 10.1111/bjso.12393 [published Online First: 2020/06/17]

38. Coronavirus: Police granted access to details of people told to self-isolate by Test and Trace. *Sky News* 2020 19/10/2020.

39. Bonell C, Michie S, Reicher S, et al. Harnessing behavioural science in public health campaigns to maintain 'social distancing' in response to the COVID-19 pandemic: key principles. *Journal of Epidemiology and Community Health* 2020;74(8):617-19. doi: 10.1136/jech-2020-214290

40. Independent SAGE. Independent SAGE statement on the new UK Government COVID Measures announced on 22nd September 2020 [updated 28/09/2020. Available from: https://www.independentsage.org/independent-sage-statement-on-the-new-uk-government-covid-measures-announced-on-22nd-september/ accessed 23/10/2020.

41. Villa S, Jaramillo E, Mangioni D, et al. Stigma at the time of the COVID-19 pandemic. *Clinical Microbiology and Infection*;https://doi.org/10.1016/j.cmi.2020.08.001 doi: 10.1016/j.cmi.2020.08.001

42. Independent SAGE, EAGLE research group for education. The Independent SAGE Report 26: An urgent plan for safer schools 2020 [Available from: https://www.independentsage.org/an-urgent-plan-for-safer-schools/ accessed 05/01/2020.

Confidential: For Review Only

Box 1: Anticipated risk reduction measures at school

The most common measures planned were:

‘Bubbles’: Groups of students (most commonly year groups) prevented from mixing with other bubbles, and often contained in one part of the school. Separate lunch and break times for bubbles.

Social Distancing: 2m distancing between teachers and students. Distancing not expected between students.

Reduced movement of students around school: students staying in one classroom and teachers moving between classrooms, staggered arrival and leaving, one-way systems, desks facing forward.

Hand sanitiser gel: available throughout school.

Cleaning: Extra cleaning/facilitating cleaning (e.g. removing soft furnishings).

Less common were:

Reduced group sessions: no/reduced assemblies or tutor groups sessions (where these were ‘vertical’ i.e. included multiple year groups).

Reduced range of subjects: suspending or adapting high Covid-19 risk lessons/activities e.g. music, cooking, science experiments, field trips.

Handwashing reminders: handwashing at certain times of day and posters/reminders to wash hands.

Increased ventilation

Handwashing facilities: Outdoor sinks.

Masks: Rules on wearing face coverings varied as the government guidance changed during data collection, but latterly schools required face coverings to be worn outside of classrooms.

l: For Review Only

Table 1 Participant characteristics

School	Deprivation quintile (IMD 2019) (1 = least, 5 most)	% of students on free school meals (2020)	% of students with English as an additional language (2018/19)	Families (n)	Staff (n)
S1	2*	under 10%	20-30%	3	5
S2	5*	30-40%	40-50%	1	3
S3	3*	10-20%	30-40%	8*	2
S4	3*	20-30%	20-30%	3	0
S5	2*	10-20%	under 10%	0	2
S6	1*	10-20%	10-20%	0	1
S7	2*	under 10%	20-30%	1	0
S8	1~	10-20%	under 10%	0	1
S9	5~	under 10%	under 10%	1	0
S10	3~	10-20%	under 10%	1	0
S11	1~	10-20%	under 10%	0	1
S12	4*	10-20%	10-20%	1	0
S13	1*	10-20%	10-20%	1	0
S14	1*	under 10%	under 10%	1*	0
Totals				20 (17 students, 20 parents) *1 family had children at both these schools	13

Table 2: Quotes on concern about risk of Covid-19 (using pseudonyms and S: School)

**Not concerned about Covid-19/risk outweighed by risk of school campuses not reopening**	**Concerns about Covid-19**
"I'm not worried about spreading it...I don't really come in contact with anyone who would be worried" (Emma, yr 8, S1)<br><br>"I'm not worried about illness [Covid-19], per se, I don't think…That's not something that worries me. I'd rather they got... got back to normal, to be honest" (Jess, Mum, S3)<br><br>"if you want the kids back at school, you, you have to accept that… there are risks associated with that" (Georgina, Mum, S3)<br><br>"Resigned acceptance [about school opening] that, that the world has changed and, um, that we kind of just have to get on with things" (Anna, SENCO, S1)	"I'm worried about um – because Jo's Dad is in the kind of very, what's the word I'm looking for? Vulnerable, like medical category 'cause he's only got one functioning lung and my Dad is…has dementia so I'm worried about [Jo] bringing coronavirus home" (Kelly, Mum, S4)<br><br>"I'm a bit concerned. Um, what precaution they're going to take and as Asian background we come on the high risk" (Fatima, Mum, S10)<br><br>"I still would feel awful if I got my partner really ill, you know, um, from my work, when he's allowed to work from home and he's, you know, following all the guidance" (Clare, Teacher, S8)<br><br>"teachers that I've spoken to that have expressed anxieties are teachers who, you know, with things like low-level asthma and, er, other conditions that put them in a position where they may not be COVID fit" (John, Teacher, S3)

Confidential: For Review Only

499 **Table 3: Quotes about school covid-19 risk reduction measures (using pseudonyms and S: School)**

**Concerns/barriers**	**Facilitators**
Social distancing impossible	Common good
"I can't see it [social distancing] happening unless there are less children in the school at one time" (Sarah, Mum, S4)<br><br>"you can't properly socially distance in a school so it's a lot of 'that'll do, that'll do' but it's still best it can be" (Matt, Teacher, S3)<br><br>"maybe they [school] have some magical plan, or maybe if they can extend something or something like that, but I... I just feel like there's not enough space for what they're trying to do" (Amber, yr 7, S3)	"I think empowering people to be autonomous and realise that their efforts are, are for the collective good. That they've all got a part to play in it. I think that's much better than, you know, er, publicly shaming them, or or, or criticising them....if you want to achieve compliance and cooperation, it's creating an atmosphere that it's in everyone's best interest and you contribute to the common good" (John, Teacher, S3)
Concerns about year bubbles	Messaging and reminders
"at the end of the day, we're all gonna walk out of school at the same time and we're all just gonna walk together and end up in the same place " (Amber, yr 7, S3)<br><br>"siblings they are mixing bubbles, the bubbles become 500, 700 [people], um, so it's a gesture" (Claire, Teacher, S8)	"if there's a consistent message, 'this is just what you do now, if you can', then, um, then they'll just pick it up really and get on with it" (Georgina, Mum, S3)<br><br>"I think having more visuals is really quite key so there's, they are reminded of what's, what's going on. Um, I don't feel like we have enough at the moment, to be honest" (Julia, Teacher, S11)
Concerns about hand-hygiene	Staff culture shift
"I suspect we're going to run out of hand sanitisers in the, um, the ones that are kind of communally put up around the school ...I'd say that they, they're going to run out by break time" (Julia, Teacher, S11)<br><br>"They [school bathrooms] never have soap or anything, like, loads of the taps don't work!" (Daisy, yr 7, S3)	"our biggest weapon will be 'don't be in work if you're ill'" (Jackie, Head, S1)
Lack of compliance with new rules	
"[on handwashing] maybe forgetting or teachers not reminding the students enough or them being too lazy or maybe their friends might be going somewhere but their	

Confidential: For Review Only

friends don't wanna wait for them so they just can't be bothered to wash their hands" (Ali, yr 10, S13)<br><br>"I feel like, you know, people are just are not gonna do it [social distance]. They're just gonna do what they want and like run around the whole place " (Amber, yr 7, S3)<br><br>"a line that's been put on the floor that shows the two metres to the teacher. Um, what's interesting there, of course some students are going to see that as a bit of an invite or a challenge to cross that line, literally" (Tracey, Mum, S3)<br><br>"[my child] might just go to the sink, wash the few fingers at the front and then leave. We don't actually know if they're going to be doing the full happy birthday… it baffles me how it's [enforcing handwashing] actually going to work" (Samira, Mum, S4)	
Specific groups	
"disadvantaged students, and students that would normally get more support in a lesson - because it's more like lecture style now, so we're not really allowed to walk between the desks or anything - those students are going to miss out, the ones who wouldn't normally put their hand up " (Finn, Teacher, S5)<br><br>"the young people … with the highest level of needs, who've got education health and care plans …might not fully understand the need for personal space, or…we've just added this whole extra kind of layer of complexity into their social world that I'm sure is going to be hugely challenging" (Anna SENCO, S1)	

Confidential: For Review Only

Table 4: Quotes about school management of Covid-19 cases

**Reactions to Covid-19 cases**
"if someone coughed in my class, I would see one or two people shying away from them a bit and that most of the class is laughing a little bit and saying, 'oh you have Corona virus' as a joke" (David, yr 8, S3)<br><br>"I think everyone would just be very pragmatic about it and I can't see there being panic stations" (John, Teacher, S3)
**Reporting symptoms to school**
"I think it will be very tricky… for parents to differentiate… fever and cough, they are quite common symptoms kids get in the winter season " (Sangita, Mum, S13)<br><br>"People may also not want to tell them about symptoms due to like embarrassment and stuff so if they – everyone finds out that they have symptoms" (Jasmine, yr 10, S4)
**School Covid-19 testing**
"If there was a risk we were going to get the virus and it would make everyone safer then I would do it [regular testing]" (Jasmine, yr 10, S4)<br><br>"I think it's good to see if people might have the coronavirus every month" (Lily, yr 8, S3)<br><br>"I understand the need [to collect data] but on the same token, it's just that personal data being collected about my child makes me feel very uncomfortable" (Sarah, mum, S4)<br><br>"market it towards the fact that they've missed so much education, this is something that can potentially help keep them in school for as long as possible. Because that's ultimately what I think most parents are concerned about" (Dan, Head of yr 8, S2)

Confidential: For Review Only

Supplementary material: Interview topic guides

School staff topic guide

1. **Demographic**

Check their role at school, how long they've been there /how long been teaching?

2. ***Experience of home learning***

Has your school been open during lockdown (for keyworker families) and what is the status now (which years are attending, approx how many of permitted children are attending etc). PROMPT: Years 8 and 10 in particular.

How have you been in contact with parents and pupils during lockdown? How much face to face teaching are you currently doing? How much are you physically in school?

What has been good / bad about teaching remotely during lockdown for you and the pupils?

What could be done/could have been done to improve the support for pupils (learning and in general) while they are at home?

Have you had feedback from parents and/or students about what they are finding difficult or what is working well?

Anything you would you like to see continue/lessons learnt that may help in the future? (PROMPTS: better communication with parents, online learning)

3. ***Attitude towards returning to school***

How do you feel about schools fully reopening in September? Why? Positives? Negatives? (PROMPTS: pupils can socialise with friends, structure to the day, educational attainment, wellbeing/mental health/anxiety, also positives and negatives for them being back at work) .

How do you feel about the government's guidance and suggested measures? Do you trust this guidance? Why do you feel this way? Is there anywhere you would prefer to get (trusted) information?

Do you have any concerns about returning to school? Examples? (PROMPTS: Concern about risk of catching coronavirus, availability of PPE, worries about maintaining social distancing/bubbles, challenges of complying with government guidance, concern about personal or family members with underlying health issues, practicalities of timetabling lessons, behaviour of pupils, supporting pupils who have fallen behind, changes in teaching compared to during lockdown (less teacher attention, expectation stay at front of class etc))

Are you concerned about any particular student groups (or staff) and why? (pupils with SEN, pupils disengaged from school/struggled during lockdown, pupils from BAME communities, pupils/teachers/staff with family members who are vulnerable

Confidential: For Review Only

to COVID-19, pupils without internet access, pupils on free school meals, particular year groups – years 8 and 10 in particular)

What is planned to support these pupils?

Are there particular groups that you feel are more cautious about sending their children back to school? How you trying to support these families?

What additional support would you like (local, national) to overcome barriers? (PROMPTS: support/information from school, availability of PPE; funding) . Is there anything that you have found helpful so far?

Have you heard from parents and/or students about their views on schools -re-opening? What are their concerns?

4. ***Attitude towards social distancing in schools***

What plans have your school put in place to facilitate social distancing? (PROMPTS: changing rooms, layout of school, changes to timetabling)

How easy / difficult do you think it will be/has been for pupils/school staff to follow social distancing in schools?

Do you have any concerns about the 'bubbles' idea and how it will work (e.g. staff crossing between bubbles)?

What could make it difficult for pupils/school staff to do this? (e.g. dedicated staff/time to do the extra work, built environment/classroom space, children remembering, fighting/bullying) Are there any particular groups of pupils that find it easier/more difficult? (older children, SEN, cultural issues, children who have been at school throughout).

What could help pupils do this? (additional classroom space / teachers, markers on the ground, verbal and written information, emphasis on reducing illness generally, reward/sanction system)

What times in the day will it be easier/more difficult for pupils to social distance from others? (PROMPTS: journey to/from school, arriving/leaving school, break and lunch times, during lessons, moving between lessons, PE lessons, in bathrooms,)

Any thoughts on whether pupils are/will be social distancing outside school?

5. ***Attitude towards hand hygiene and infection control strategies in schools***

What policies have been put in place to encourage increased of frequency and thoroughness of handwashing by students? How about touching of the face? Ventilation (SAGE advice hourly)? How about cleaning in school? Any other infection control measures? Any guidance on masks?

So far what has been the students' attitude to handwashing ?

Impact of new handwashing & hygiene practices on time dedicated to learning?

Confidential: For Review Only

How easy / difficult is it and do you think it will be for pupils/school staff to wash hands more regularly in schools?

What could stop pupils/school staff doing this? (already doing as much as they can, availability of hand washing facilities, bullying in bathrooms)

What has helped and could help pupils/school staff do this? (behaviour change intervention, verbal and written reminders, handwashing incorporated into timetabling, additional facilities, provision of hand sanitisers)

What times in the day will it be easier/more difficult to do this?

6. ***Impact on learning***

Do you have any comments on how you think lockdown/school closure/social distancing in schools has/will affect pupil's learning? How will your teaching methods need to change?

What could help to improve this/reduce any negative impact? Guidance, funding, etc?

7. ***Plans for infections***

Does the school have a plan for dealing with any suspected/diagnosed Covid infections? (keeping kids off school, reporting mechanisms, testing, contact tracing, school closure)? Will you increase risk reduction measures (in line with recent SAGE advice) e.g. masks, smaller bubbles, no group working?

Would there be any issues if a class or year group needed to self-isolate? How would you feel about having to self-isolate (for at least 7 days) if pupils /you/other staff developed the symptoms of coronavirus? Negatives? Positives?

Is it possible for you to self-isolate?
What could stop you self-isolating?
What could help you self-isolate?

What is the biggest issue if a whole school local lockdown is required, with pupils to remain at home? Why? If school lockdown happens, school will have to provide remote education support – what would the school provide and would there be any challenges?

8. ***acceptability of possible new school test-and-trace study to map Covid-19 infections in students and staff using saliva sampling***

Researchers at the University of Bristol are developing a possible new study. This study will help understand how the virus spreads among young people and in schools. The study will involve testing young people in certain school years *and school staff for coronavirus using saliva sampling system (taken yourself) once a month for 6 months. It would also require reporting of symptoms to the school. Infection will be linked to home postcode and linked to NHS data to understand who is getting*

Confidential: For Review Only

the virus and where. A system will be developed and tested that uses different sources of information to enable contact tracing and help control outbreaks.

How would you feel about a mapping study like this in your school? Positives? Negatives? Is testing once a month feasible? Test will be spitting into a tube, would there be any problems you all doing this together in public in your tutor group? – why? How overcome?

Anything that would make it difficult or easier? What would be an incentive for your school to take part?

Are there any issues specific to years 2,5,8,10,12?
Any suggestions on how we should present it to young people/families?
How to alert individuals, classes, school of suspected cases?
How best to provide tests? When? In classrooms, take home kits, school nurses office?
How to take consent? Student consent ?

How would you feel if you tested positive? Or if someone in the school did? Stigma if pupils/school staff develop symptoms, if outbreak ripples into community, concern for he alth

If there was a mapping study like this one in your school and you developed symptoms, how likely is it that you would report them? Same question for pupils/other staff?

What would be the most important reason you /pupils/other staff would decide to report symptoms of coronavirus?

What would be the most important reason that would stop pupils/you/other staff from reporting symptoms of coronavirus? (PROMPTS: stigma, bullying, data protection concerns)

The study would also provide an intervention to help reduce the risks of Covid, which may be a modified version of Germ Defence (Germ Defence is a website that provides advice on infection control measures and helps users think about when and how to carry out key infection control behaviours such as handwashing and cleaning, avoiding sharing rooms and surfaces, managing incoming deliveries, and ventilating rooms. Behaviour change techniques are used to help people implement this advice).

What might be useful for school to help them implement new measures/interventions like this?

Confidential: For Review Only

Parent/Guardian topic guide

1. Background question
How old is your secondary school aged child(ren)?
What area of Bristol do you live in?
[may need to ask this at the end without child] Do you have any particular concerns about Covid-19?

2. ***Experience of home learning – don't spend too long on this section***
Has your child(ren) been into school during the lockdown? **N.B. Tailor subsequent questions depending on whether child has been into school.**

What has been good / bad about your son/daughter being out of school during lockdown? Has there been an emotional impact of social distancing/isolation/not attending school?

What could be done to improve your / your son/daughter's experience of home learning or other things they are missing from school?

Anything you would like to see continue?

What is the biggest issue if a whole school local lockdown is required, with pupils to remain at home? Why? If school lockdown happens, school will have to provide remote education support - when teaching pupils remotely, what would you expect the schools to provide to support home learning as a minimum ? Why ?

3. ***Attitude towards returning to school***
How do you feel about your son/daughter returning to school in September? Positives? Negatives? (PROMPTS: support parents to work, young person can socialise with friends, structure to the day, educational attainment, wellbeing)

Where has your information about school reopening come from - school, council, government, other parents? Which information would you trust the most when thinking about going back to school and making decisions around this?

Do you have any concerns about your son/daughter returning to school? Examples? (PROMPTS: Concern about risk of catching coronavirus, worries about maintaining social distancing, concern about family members with underlying health issues, pressures of work/catching up). Is there anything particular about their school year/age?

What would like the school to do to help address your concerns? (PROMPTS: support/information from school)

What additional support would you like (local, national) to overcome concerns ?

4. ***Attitude towards social distancing in schools***
How has your child(ren) found social distancing so far?

Confidential: For Review Only

Do you think the school need to do things like social distancing and hand washing? Why/why not?

Do you know what plans the school has to enable social distancing in schools? (PROMPTS: changing rooms, layout of school, limiting number of children in school, a mix of online and face to face teaching)

The government has also brought in the idea of year bubbles, what are your views on that? Do you think it will work? Will it work outside of school?

How easy / difficult do you think it will be for young people to follow social distancing in schools?

What could stop young people from doing this? (e.g. built environment/classroom space, fighting/bullying, peer pressure)

What could help young people do this? (additional classroom space / teachers, markers on the ground, verbal and written information)

What times in the day will it be easier/more difficult for young people to social distance from others? (PROMPTS: commute to school, arriving/leaving school, break and lunch times, during lessons, moving between lessons, PE lessons, in bathrooms, after school, weekends)

Do you think social distancing measures will affect learning?

5. ***Attitude towards hand hygiene in schools***

How has your child(ren) found following hand washing advice so far? Do you have any concerns about possible negative impact of extra hand-washing e.g. compulsive behaviour or less exposure to germs? Anything that could be done to address these concerns?

How important do you think hand washing is at school (re Corona)? Do you know what the school is putting in place to encourage frequency and thoroughness of handwashing by students ?

How easy / difficult do you think it will be for young people to wash hands in schools?

What could stop young people doing this? (availability of hand washing facilities, bullying in bathrooms)

What could help young people do this? (verbal and written reminders, handwashing incorporated into timetabling, additional facilities, provision of hand sanitisers)

What times in the day will it be easier/more difficult to do this?

6. ***Acceptability of test-and-trace study***

Researchers at University of Bristol are developing a possible new school test-and-trace study to map Covid-19 infections in students and staff This study will help understand how the virus spreads among young people and in schools. The study will involve testing young people and school staff for coronavirus using saliva sampling (taken yourself) once a month for 6 months, and reporting symptoms to school.. infection will be linked to home postcode & linked to NHS data to understand who is getting the virus & where. A system will be developed and tested that uses different sources of information to enable contact tracing and help control outbreaks.

Confidential: For Review Only

How would you feel about a mapping study like this in your child's school? Positives? Negatives?

Only positive test results would be sent out (no news = good news) – would you have any concerns about this? Also positive test results will be sent to Headteacher, Bristol City Council Health Protection Team & nationally Public Health England – do you have any issues with this? Why?

Test will be spitting into a tube, would there be any problems everyone doing this together in public in your tutor group? – why? How overcome? If there is a confirmed case from school testing – all members of household/contacts will be asked to provide weekly samples for 4 weeks – how would you feel about this? Any problems with this? Why? How to do this without causing issues/concerns

Would you have any concerns about your child taking part in a test and trace system like this? What would help allay those concerns?

What age do you think it would be appropriate for children to give their own consent for this? How would you feel about your child(ren) giving their own consent? Why?

If there was a mapping study like this, how likely do you think it would be that children at school would report symptoms of coronavirus? What would be the barriers/facilitators?

How would you feel about your family having to self-isolate (for at least 7 days) if you/your child developed the symptoms of coronavirus? Negatives? Positives?

Do you have the space at home for your child to self isolate?

How would you feel about the whole family having to self-isolate if someone you/your child had been in contact with developed the symptoms of coronavirus?

Confidential: For Review Only

Young person's topic guide

1. ***Experience of lockdown and education– don't spend too long on this section***

Have you been into school during the lockdown? **N.B. Tailor subsequent questions depending on whether they have been into school.**

What has been good / bad about home learning (e.g. learning remotely) during lockdown? What have you missed/not missed about school? How have you been feeling during this time?

What has helped during this time?

What could have the school done to help make it easier?

How do you think home learning has affected your learning?

PROMPTS : What could be done to improve experience of home learning? What would you like to keep doing?

2. ***Attitude towards returning to school***

How do you feel about returning to school in September? Positives? Negatives? (PROMPTS: socialising with friends, structure to the day, education, wellbeing, concern about risk of catching coronavirus, worries about maintaining social distancing)

Where has your information about school reopening come from - school, council, government, other parents? Which information would you trust the most when thinking about going back to school and making decisions around this?

Do you have any concerns or worries about returning to school? Examples? (PROMPTS: Concern about risk of catching coronavirus, worries about maintaining social distancing, concern about family members with underlying health issues, peer relationships)

What would help you feel better about these worries or what would help solve those worries? (PROMPTS: support/information from school)

Do you think your learning will be the same?

Are you aware of the school's plans for future lockdown/2nd wave/local lockdown? How do you feel about these plans e.g. a mix of online and face to face teaching?

3. ***Attitude towards social distancing in schools***

How have you found social distancing so far (keeping 2m apart from people)? How much do you think people your age have followed social distancing when they are out?

What has made it difficult to social distance? What could make it easier?

Do you think the school need to do things like social distancing and hand washing? Why/why not?

How easy / difficult do you think it will be to follow social distancing (stay 2 metres apart from others) in schools?

Confidential: For Review Only

To help with social distancing, the government has also brought in the idea of year bubbles, where you are asked to stay in your class/year group and not mix with other outside it, what are your views on that? Do you think it will work? Will it also work outside of school?

What could stop you from doing this? (e.g. built environment/classroom space, fighting/bullying/wanting to feel close to friends again)

What could help you do this? (additional classroom space / teachers, markers on the ground, verbal and written information) do you know what the school is planning to make it easier for you?

What times in the day will it be easier/more difficult to social distance from others? (PROMPTS: journey to/from school, arriving/leaving school, break and lunch times, during lessons, moving between lessons, PE lessons, in bathrooms, after school and at weekends)

Do you think the changes at school will affect your learning?

4. Attitude towards hand hygiene in schools

What have you been told so far about washing your hands? How important do you think hand washing is at school (re Corona)?

Do you know what your school is putting in place to encourage and handwashing ?
How easy / difficult do you think it will be to wash hands more regularly in schools?

What could stop you doing this? (availability of hand washing facilities, bullying in bathrooms)

What could help you do this? (verbal and written reminders, handwashing incorporated into timetabling, additional facilities, provision of hand sanitisers)

What times in the day will it be easier/more difficult to do this?

5. Acceptability of test-and-trace study

Ascertain if they know much about the national test and trace scheme and tailor this description accordingly. Researchers at the University of Bristol are developing ways to test young people and school staff for coronavirus using saliva samples (you spit into a container). This study will help them understand how many people have the virus and how it gets passed on. Where the virus is found it will be linked to the person's home postcode and linked to a NHS database to help see which people have the virus and where they live. The researchers would also look at ways to trace who that person had been mixing with. This information may help control the virus and stop outbreaks.

How would you feel about a test and trace study (like the one I've just described) in your school? Positives? Negatives? Is testing once a month feasible?

What would be the best way to take saliva samples in school? In tutor groups, classrooms, take home kits, school nurses office? Test will be spitting into a tube, would there be any problems you all doing this together in public in your tutor group? – why? How overcome?

Only positive test results would be sent out (no news = good news) – would you have any concerns about this? What would be the best way to be told if you tested positive? Also positive test results

Confidential: For Review Only

will be sent to Headteacher, Bristol City Council Health Protection Team & nationally Public Health England – do you have any issues with this? Why?

If there is a confirmed case from school testing, all members of household/contacts will be asked to provide weekly samples for 4 weeks – how would you feel about this? Any problems with this? Why? How to do this without causing issues/concerns?

Do you know anyone who has had a positive Corona test? (if not then ask hypothetically if they/someone else did test positive). Do you think that person minded/would mind other people knowing or were/might be treated unkindly by anyone or how they felt /might feel about it? Why? PROMPTS: Stigma, concern about health, concern about having to stay home again

How would you feel about people tracing who you had been mixing with?

If there was a test and trace study like this one in your school and you developed coronavirus symptoms, how likely is it that you would report them?

Why would you decide to report symptoms of coronavirus?

Why might you decide not to report symptoms of coronavirus? (PROMPTS: stigma, bullying, data protection concerns, make sure cover concerns about testing and also concerns about being diagnosed)

How would you feel about having to self-isolate (for at least 7 days) if you developed the symptoms of coronavirus or tested positive? Negatives? Positives?

Do you have the space at home to self isolate?

What could stop you self-isolating?

What could help you self-isolate?

How would you feel about having to self-isolate if someone you had been in contact with developed the symptoms of coronavirus?

Confidential: For Review Only
